# On the Fine Isotopic Distribution and Limits to Resolution in Mass Spectrometry

**DOI:** 10.1007/s13361-015-1180-4

**Published:** 2015-08-12

**Authors:** Piotr Dittwald, Dirk Valkenborg, Jürgen Claesen, Alan L. Rockwood, Anna Gambin

**Affiliations:** Institute of Informatics, University of Warsaw, Warsaw, Poland; Applied Bio and Molecular Systems, VITO, Mol, Belgium; Center for Proteomics, University of Anwerp, Antwerp, Belgium; Interuniversity Institute for Biostatistics and Statistical Bioinformatics, Hasselt University, Hasselt, Belgium; Department of Pathology, University of Utah School of Medicine, Salt Lake City, UT 84132 USA; ARUP Laboratories, a Nonprofit Enterprise of the University of Utah, Salt Lake City, UT 84108 USA; College of Inter-Faculty Individual Studies in Mathematics and Natural Sciences, University of Warsaw, Warsaw, Poland

**Keywords:** fine isotopic distribution, Thermorelativistic effect, Limits to resolution, Mathematical modeling

## Abstract

Mass spectrometry enables the study of increasingly larger biomolecules with increasingly higher resolution, which is able to distinguish between fine isotopic variants having the same additional nucleon count, but slightly different masses. Therefore, the analysis of the fine isotopic distribution becomes an interesting research topic with important practical applications. In this paper, we propose the comprehensive methodology for studying the basic characteristics of the fine isotopic distribution. Our approach uses a broad spectrum of methods ranging from generating functions—that allow us to estimate the variance and the information theory entropy of the distribution—to the theory of thermal energy fluctuations. Having characterized the variance, spread, shape, and size of the fine isotopic distribution, we are able to indicate limitations to high resolution mass spectrometry. Moreover, the analysis of “thermorelativistic” effects (i.e., mass uncertainty attributable to relativistic effects coupled with the statistical mechanical uncertainty of the energy of an isolated ion), in turn, gives us an estimate of impassable limits of isotopic resolution (understood as the ability to distinguish fine structure peaks), which can be moved further only by cooling the ions. The presented approach highlights the potential of theoretical analysis of the fine isotopic distribution, which allows modeling the data more accurately, aiming to support the successful experimental measurements.

**Electronic supplementary material:**

The online version of this article (doi:10.1007/s13361-015-1180-4) contains supplementary material, which is available to authorized users.

**Graphical Abstract:**
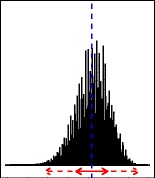
ᅟ

## Introduction

An important challenge in mass spectrometry (MS) is to extend the technology to enable detection of large organic particles, biomolecules, and nano-particles. Several solutions have already been proposed to analyze very large particles, such as modified quadrupole TOF (QTOF) tandem mass spectrometer or charge detection mass spectrometry (CD-MS) [1]. These two main approaches compete with each other in respect of MS analysis: multiple charging followed by an appropriate charge detection phase and trapping system that can be used to sample singly charged massive ions. Wang et al. [2] state that at least for the latter approach, there is now essentially no mass limit. In the CD-MS technology, the mass resolution depends strongly on the quality of the charge measurement, but we are not aware of any fundamental obstacle standing in the way to improve mass resolution.

In this paper, we argue that in fact there exist the impassable limits to mass resolution; however, our main goal is to provide the theoretical framework to investigate these constraints. Although very theoretical in its nature, the developed methodology could be useful in experimental practice. There are already examples of successful assays of very heavy biomolecules (e.g., viral assemblies in megadalton (MDa) mass range [1, 3, 4]). Therefore, the investigation of theoretical limits for the applicability of the isotopic distribution, especially for heavy particles, can be useful to the mass spectrometry community for improving experimental design and data processing. This could help to avoid planning experiments that will be unable to succeed because of these limits, or to plan experiments with the purpose of overcoming these limits [5].

The evolution of isotopic distribution calculations based upon the molecular formula and elemental isotopic distribution (c.f. Supplementary Table 1) in terms of accuracy and speed harmonizes with the increasing resolution of mass spectrometers. However, how an isotope profile is displayed by mass spectrometry depends to a large extent on the effective resolution of the instrument to resolve the isotope variants. In this sense, Fourier transform MS (FT-MS) differs from lower resolution instruments like ion traps, time-of-flight MS, etc.

**Figure 1:**
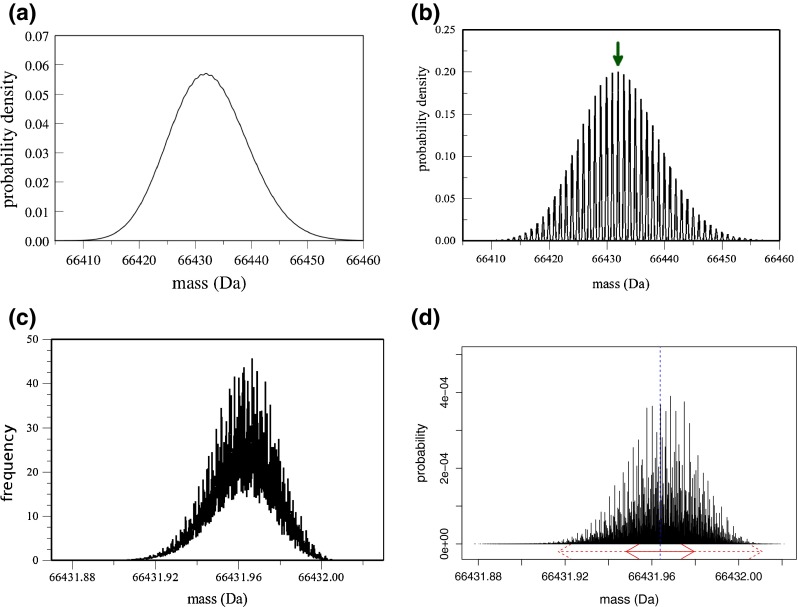
**(a)** Isotopic distribution of the bovine serum albumin (*C*_2934_ *H*_4615_ *N*_781_ *O*_897_ *S*_39_) with low resolution. **(b)** The same distribution as in **(a)**, but with higher resolution that reveals the individual aggregated variants. Green arrow indicates the most abundant aggregated variant (42 additional neutrons wrt the monoisotopic variant). **(c)** Profile of the fine structure of the most abundant aggregated variant with high resolution. Data for **(a)**–**(c)** use calculations performed with mercury [6, 7] with increasing resolutions 49,600 and 248,000 and 305,000,000 FWHM, respectively. These plots are normalized such that the area under the curve sums up to 1. **(d)** Stick representation of the fine structure of the most abundant aggregated peak of bovine serum albumin obtained using isoDalton [8] software with parameters set to 1,000,000 most abundant fine peaks. The dotted blue line marks the center mass (i.e., the mean of the fine structure) as extracted from data. The red arrows show the interval that is ±*σ* (continuous line) or ±2*σ* from the center mass (center mass and σ are calculated using the moment generating function approach - see the Methods section)

In the latter case, the resolving power is usually large enough to baseline separate the isotope profile of intact and multiple charged molecules such that distinct peaks with 1 Da mass differences are observed, whereas in low resolution mass spectrometry the isotope profile of a large molecule is presented as a Gaussian shaped peak profile. For example, the isotope distribution of bovine serum albumin (*C*_2934_*H*_4615_*N*_781_*O*_897_*S*_39_) is depicted in Figure 1 at different levels of resolution. Figure 1a presents the aggregated isotope distribution at a resolution of 49,600 FWHM. It can be noted that the baseline-resolved peaks start to fuse into a Gaussian-like shape.

On the other hand, Figure 1b displays the baseline resolved isotope profile of Figure 1a, but at a resolution of 248,000 FWHM, where peaks appear with a mass difference of approximately 1 Da. It should be noted that these peaks assemble various isotope variants with the same nucleon count, yet slightly different masses. Therefore, we denote these peaks as aggregated isotope variants. In order to disassemble an aggregated isotope variant into its fine isotope structure, high resolving power is required (e.g., FT-MS). A theoretical illustration of the isotope fine structure at infinite resolution is depicted in Figure 1d for the aggregated isotope variant denoted by the arrow in Figure 1b. The distribution in Figure 1c looks similar as the one in Figure 1a, however, should not be confused. Where Figure 1a illustrates the low resolution profile of an aggregated isotope distribution, the peak in Figure 1c is a single aggregated isotope variant at a higher resolution, such that abundant fine structure variants appear as shoulders on the peak shape. It is also worth noting that in Figure 1c, even a resolution of >300,000,000 FWHM is not sufficient to baseline-resolve the isotope structure of the aggregated isotope variant denoted by the arrow on Figure 1b.

**Figure 2:**
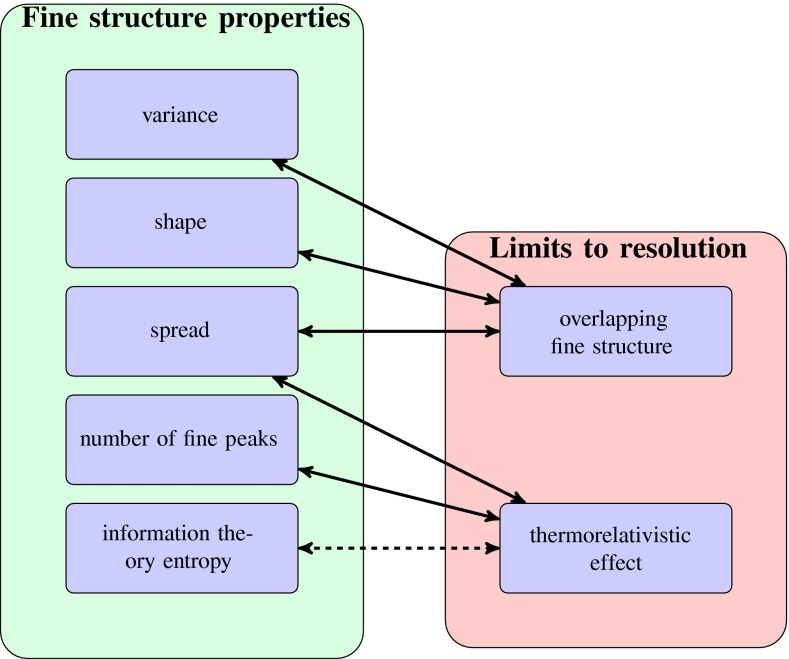
Overview of the results presented in the paper: theoretical methods to investigate the fine structure distribution (left) and how they illustrate the limitations of MS data analysis with respect to the mass resolution (right). Arrows indicate the related concepts, dotted line shows the relationship for further investigation (see the end of “How would thermorelativistic effects influence isotope resolution?”)

In this manuscript, we focus on some properties of the isotope fine structure such as shape, spread, and variance as displayed in Figure 2. The methods we use to infer these properties vary from extending moment generating functions [9, 10] to using concepts rooted in information theory (entropy), and investigating “thermorelativistic” effects, i.e., mass uncertainty attributable to relativistic effects coupled with the statistical mechanical uncertainty of the energy of an isolated ion (please note, we will from here on use the word “thermorelativistic” without quotation marks). While discussing our methodology, we consider two applications where the developed theory could be helpful, namely, modeling the fine structure distribution, and investigating the limits of molecular fine structure in real experiments.

## Methods

This section briefly introduces the various tools developed for characterizing the aggregated isotope distribution and isotope fine structure. “Polynomial generating function” introduces the polynomial function as used in the BRAIN algorithm [10, 11]. “Variance of the fine isotopic distribution of an aggregated isotopic variant” describes how this polynomial can be extended to calculate the second moment, (i.e., the variance). “Information theory entropy of the fine structure of an aggregated isotopic variant” explains the calculation of information theory entropy based on the polynomial generating function. Finally, “Maximum spread of the fine structure of an aggregated isotopic variant” and “Number of fine peaks of an aggregated isotopic variant” present predictive models to estimate the spread and the number of fine peaks of a given aggregated variant, respectively.

### Polynomial Generating Function

In this article, we consider only substances with a chemical formula of *C*_*v*_*H*_*w*_*N*_*x*_*O*_*y*_*S*_*z*_. Note that extending this approach to more elements is straightforward.

First, we introduce the polynomial used, e.g., by Claesen et al. [10] to explicitly identify isotopic peaks with the same nucleon number:

1 $$ Q\left(I;\ v,w,\ x,\ y,\ z\right) = {\displaystyle \sum_j{q}_j{I}^j} $$

where *q*_*j*_ is a probability of *j*-th aggregated isotopic variant of the considered molecule *C*_*v*_*H*_*w*_*N*_*x*_*O*_*y*_*S*_*z*_ (i.e., variant with j additional neutrons in comparison with the “monoisotopic” variant, taken in this paper to mean the isotopic peak composed of all-light elemental isotopes, or alternatively refering to the aggregated isotopic variant as the isotopic fine structure cluster), and *I* is an indicator variable.

Recall that the center-mass (i.e., expectation value for mass) for the *j*-th isotopic variant, *m*_*j*_, is defined as:

2 $$ E\left({m}_j\right)=\frac{{\displaystyle {\sum}_k{m}_{jk}{p}_{jk}}}{{\displaystyle {\sum}_k{p}_{jk}}} $$

where *m*_*jk*_ and *p*_*jk*_ are, respectively, masses and probabilities of the *k*-th isotopic fine variant contributing to the *j*-th isotopic aggregated variant (as defined above). Equation (2) calculates the expected center-mass value as the weighted sum of masses of fine variants (the denominator normalizes an aggregated variant to the unit area). Hence, we are taking a localized view on this single aggregated variant, considered as a distinct probability distribution, rather than being part of the full isotope distribution.

### Variance of the Fine Isotopic Distribution of an Aggregated Isotopic Variant

We study here the second moment of the center mass variable, which illustrates the variability of the fine structure relative to the center mass values. Combined with the first moment (i.e., the mean of the center mass values) the variance provides substantial information on the distribution of the fine isotope structures, without the requirement of explicitly calculating the fine isotope variants. The variance can be calculated as follows:

3 $$ \mathrm{V}\mathrm{a}\mathrm{r}\left({m}_j\right) = E\left({m}_j^2\right)-E{\left({m}_j\right)}^2 $$

*E*(*m*_*j*_ )^2^ can be easily calculated from Equation (2) (see also e.g., [10, 12] for the algorithms to obtain this value effectively). Analogously, *E*(*m*_*j*_^2^) can be calculated using the second order derivative polynomial generating functions with the use of the second derivative (see Appendix for details).

Besides the variance of the single aggregated variants, we can also consider the total variance of the isotopic distribution that includes the fine structure (for more details see Appendix):4where the average mass of the total isotopic distribution is denoted by $\overline{m}$. This result corresponds to the well-known statistical method analysis of variance (ANOVA), which distinguishes between within-group and between-group variability. However, it should be underlined that the total variance of the distribution is composed of weighted within-variant variance (♡), and the variance of the aggregated isotopic distribution (♢).

### Information Theory Entropy of the Fine Structure of an Aggregated Isotopic Variant

The information theory entropy can be used as a measure for the complexity of an isotopic fine structure cluster (i.e., isotopic aggregated variant). If an isotopic peak is composed of only a single fine structure peak, the information theory entropy is zero. Otherwise it is a positive number, rising as the complexity of the isotopic fine structure cluster increases. The information theory entropy for the *j*-th isotopic aggregated variant can be computed from the formula (see Appendix for details):

5 $$ H(j) = \frac{-{\displaystyle {\sum}_k{p}_{jk} \log \left({p}_{jk}\right)}}{{\displaystyle {\sum}_k{p}_{jk}}}+ \log \left({\displaystyle \sum_k{p}_{jk}}\right). $$

Note that Equation (5) consists of a fractional and logarithmic component. Under the logarithm, we have probabilities of an aggregated variant, so they can be calculated using e.g., the original BRAIN algorithm. Moreover, the structure of the fractional component in Equation (5) is analogous to Equation (2) (”*m*_*jk*_” is replaced by “− log *p*_*jk*_”). We can simply use the algorithms to calculate the center-masses (e.g., [10, 12]), but replace the masses of the elements by the negative logarithm of their probabilities followed by adding the logarithm of probabilities for each aggregated variant.

Additionally, it should be noted that one can also consider the information theory entropy *H* of the full isotopic distribution, including the fine isotopic structure (detailed calculations in Appendix):6

Of note, the total information theory entropy *H* that includes the fine structure can therefore be split into two parts: the weighted sum of the information theory entropies of the fine distributions within aggregated variants (♣), and information theory entropy of the total aggregated isotopic distribution (♠) (fine structure excluded).

### Maximum Spread of the Fine Structure of an Aggregated Isotopic Variant

Here, the main interest is to identify the minimal and maximal mass value in the fine isotopic distribution of a given aggregated isotopic variant. In order to identify these two values, we calculate the mass-per-additional-neutron ratio for isotopic variants with at least one extra neutron (Supplementary Table 1). The lower and upper limit for mass increase per neutron is found for ^15^ *N* and ^2^*H*, respectively. We will denote these values as μ_*2H*_ and μ_*15N*_. The lightest fine isotopic variant only contains additional neutrons from nitrogen, whereas for the heaviest variant only additional neutrons from hydrogen can be found. Hence, the theoretical fine isotopic structure spread for an aggregated isotopic variant with *i* additional neutrons of molecule *C*_*v*_*H*_*w*_*N*_*x*_*O*_*y*_*S*_*z*_ can be calculated:

7 $$ \mathrm{Spread} = i\times \left({\mu}_{2H}-{\mu}_{15N}\right). $$

It should be noted here that in theory it is possible that the maximum observable spread will be smaller than the theoretical maximum spread because of a lower number of available hydrogens and nitrogens (i.e., when *i* > min(*w, x*)). As such, Equation (7) can be considered to be an upper bound of the theoretical maximum spread.

### Number of Fine Peaks of an Aggregated Isotopic Variant

In order to estimate the size of the isotopic fine structure for a given aggregated variant, we count the number of fine peaks in its mass spectrum. We distinguish between two fine isotopic peaks if and only if the number of isotopes for any chemical element of a given molecule is different. For example, the aggregated isotopic variant of molecule *C*_*v*_*H*_*w*_*N*_*x*_*O*_*y*_*S*_*z*_, which has one additional neutron compared with the monoisotopic variant, consists of five fine isotopic peaks (for simplicity in this subsection we assume that min(*v, w, x, y, z*) ≥ 2), i.e.,

$$ \begin{array}{l}{}^{12}{C}_v{{}_{-}}_1{}^{\mathbf{1}\mathbf{3}}\boldsymbol{C}_{\mathbf{1}\ }{}^1H_w{}^{14}N_x{}^{16}O_y{}^{32}S_z,\hfill \\ {}{}^{12}{C}_v{}^1H{{}_w}_{-1}{}^{\mathbf{2}}\boldsymbol{H}_{\mathbf{1}}{}^{14}N_x{}^{16}O_y{}^{32}S_z,\hfill \\ {}{}^{12}{C}_v{}^1H_w{}^{14}N{{}_x}_{-1}{}^{\mathbf{1}\mathbf{5}}\boldsymbol{N}_{\mathbf{1}}{}^{16}O_y{}^{32}S_z,\hfill \\ {}{}^{12}{C}_v{}^1H_w{}^{14}N_x{}^{16}O{{}_y}_{-1}{}^{\mathbf{1}\mathbf{7}}\boldsymbol{O}_{\mathbf{1}}{}^{32}S_z,\hfill \\ {}{}^{12}{C}_v{}^1H_w{}^{14}N_x{}^{16}O_y{}^{32}S{{}_z}_{-1}{}^{\mathbf{33}}\boldsymbol{S}_{\mathbf{1}}.\hfill \end{array} $$

The second aggregated peak has two additional neutrons from one of the 17 following combinations of isotopes (the remaining atoms occur as their lightest isotope): ^18^*O*, ^34^*S*, ^13^*C*^13^*C*, ^2^*H*^2^*H*, ^15^*N*^15^*N*, ^17^*O*^17^*O*, ^33^*S*^33^*S*, ^13^*C*^2^*H*, ^13^*C*^15^*N*, ^13^*C*^17^*O*, ^13^*C*^33^*S*, ^2^*H*^15^*N*, ^2^*H*^17^*O*, ^2^*H*^33^*S*, ^15^*N*^17^*O*, ^15^ *N*^33^*S*, ^17^*O*^33^*S*. We observe that the number of fine peaks increases drastically with the number of additional nucleons.

To solve the problem, we formulate this as a variant of the classic Money Exchange Problem (i.e., for a given set of coins with different denominations, find all possible combinations of these coins that sum up to a given value). In the setting of the fine isotopic peaks of an aggregated variant, the denominations correspond to the number of additional neutrons in stable isotope, i.e.

$$ \left\{{}^{13}C,{}^2H,{}^{15}N, {}^{17}O,{}^{18}O,{}^{33}S,{}^{34}S,{}^{36}S\right\}=\left\{1,\;1,\;1,1,2,1,2,4\right\} $$

and the sum corresponds to the total number of additional neutrons in the aggregated variant. The Money Exchange Problem can be solved (e.g., using a naive and inefficient implementation which enumerates all variants). Alternatively, one might consider dynamic programming approaches or the extended-round-robin-algorithm in conjunction with the “extended residue” table as implemented in decomp software [13]. Supplementary Figure 1 shows that the number of fine peaks for four atoms (C, H, N, O) increases faster than linearly. However, we should emphasize that many fine isotopic peaks will be too small to be distinguishable from noise peaks.

## Results

As already mentioned, we illustrate how the above methodology could be used in two applications. First, a predictive model to compute distributional characteristics is introduced. Second, a thorough analysis of the fine isotope distribution is conducted in view of their statistical properties (e.g., normality, etc.). This section contains also the visual exploration of the isotope distribution for selected peptides and proteins. We calculate theoretical characteristics of their fine isotopic distributions (variance and entropy), see also Supplementary Figures 5 and 6. Other interesting features such as the spread and the number of fine peaks are estimated as well. Finally, we investigate the discrepancy between the studied fine structures and the normal distribution.

To visualize and to validate the presented methodology, we applied mercury, a tool to calculate with ultrahigh resolution the fine isotopic distribution of an aggregated variant (see [7] and Appendix “Brief overview of ultrahigh resolution using the mercury Program”).

### Can we Estimate Peak’s Fine Structure Variability Based on its Center-Mass?

We investigated whether the variance of the isotope fine structure varies over the mass. Therefore, we processed around 58,000 proteins (each chemical formula is used only once) from the UniProt [14] database and calculated the variance of the most abundant aggregated isotope peak.

**Figure 3:**
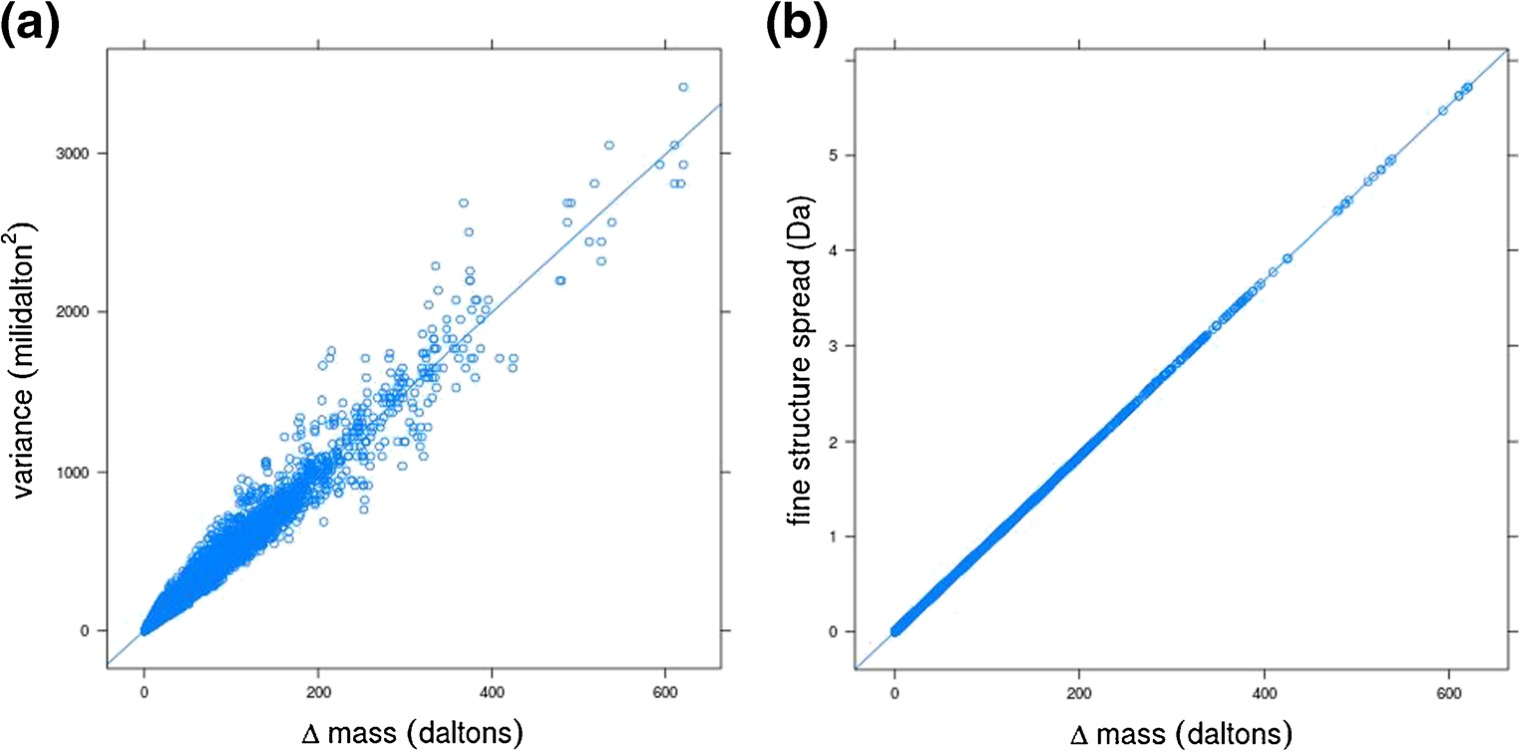
The relationship between variance **(a)** and spread **(b)** of the most abundant peak versus Δm (the mass difference between this variant and the monoisotopic one) for the proteins from Uniprot database. In addition, the regression lines were plotted. On **(b)** we use a conservative estimate that looks at mass difference of additional neutron between nitrogen and hydrogen (for proteins with most abundant peak with 10 additional neutrons we can assume we have at least 10 hydrogens and 10 nitrogens). Note that the spread covers also very tiny peaks of the fine structure, as plotted in Figure 1. Therefore the variance-based estimate (e.g., 6*σ*) is a much more realistic approach to capture the actual fine structure width

Note that Figure 3a illustrates the variance as a function of ∆*m* (i.e., difference between the mass of the most abundant peak and the mass of the monoisotopic peak). However, given the fact that the most abundant peak is a nearly linear function of the molecular weight (especially at high molecular weight) a qualitatively similar linear relationship is obtained for the dependence of variance on the molecular weight of the protein. Having this linear relationship observed, we built a simple linear regression model linking the variance of the most abundant aggregated isotope peak, denoted by Var(*m*_*a*_), to its center-mass (*m*_*a*_):

8 $$ \mathrm{V}\mathrm{a}\mathrm{r}\left({m}_a\right) = 1.503\times {10}^{-6} + 3.077\times {10}^{-9}\times {m}_a. $$

Additionally, we checked in the UniProt database the maximum theoretic spread of the fine structure as introduced in “Maximum spread of the fine structure of an aggregated isotopic variant”. This spread can be larger than 1 Da for some biomolecules (cf. Figure 3). However, the variance of the fine isotopic distribution is small, suggesting a large number of fine isotopic peaks with a high probability close to the center mass of the aggregated isotope variant.

### When do Isotopic Fine Structures Overlap?

Next, we identified the approximate size (in Daltons) of a molecule for which the overlap between consecutive aggregated peaks may happen. More precisely, we wanted to predict the center mass of the most abundant aggregated variant for which the standard deviation within this variant reaches 0.5 Da. We assumed that the distribution of isotopic fine clusters have a Gaussian shape. As a consequence of this assumption, a substantial part of their distribution will not be part of the interval $\overline{m}\pm \sigma$, where $\overline{m}$ and *σ* are its mean and standard deviation of the normal distribution, respectively. Based upon this assumption, we checked for which most abundant center mass (Equation (8)), the standard deviation is equal to or larger than 0.5 Da (cf. Supplementary Material). The predicted mass, where the overlap between adjacent isotopic fine structure clusters starts is ∼ 81.25 MDa.

However, despite the fact that 81.25 MDa is much larger than the proteins in the Uniprot database, this molecular weight range is not as hypothetical as it might seem. Nearly two decades ago, Chen et al. [15] demonstrated the trapping and detection of coliphage T4 DNA ions with a molecular weight of 110 MDa. With a method similar to that used to estimate the variances of isotopic peaks of peptides, the variance of a base peak of large DNA molecules can be estimated from an equation analogous to Equation (8) with the molecular weight multiplied by 1.68 × 10^−9^ Da. This corresponds to a standard deviation of 0.43 Da for the coliphage DNA observed in [15]. Consecutive fine isotopic peaks with such a wide spread will be overlapping heavily and, as such, difficult to resolve, as illustrated in Supplementary Figure 3A.

By comparison, the aggregated isotopic envelope (including all isotopic peaks) of a DNA molecule of 110 MDa would have a standard deviation of ∼249 Da or a FWHM of approximately 586 Da (see Supplementary Figure 5). A mass spectrometer with a resolution of ∼5 × 10^5^ FWHM would be sufficient to resolve the overall isotopic envelope and, as a practical matter, a mass spectrometer of this resolution would reveal most of the information available.

A more recent paper [2] demonstrated the trapping of singly charged urea particles of molecular weights as high as 3 GDa. This work was presented as a major step toward high resolution mass analysis of RNA, DNA, and viruses. If we assume that the variance of an isotopic peak of an intact virus particle would scale as 2.4 × 10^–9^ times the molecular weight (approximately the average of the scaling factors for proteins and DNA), the isotopic peaks of a virus particle in this molecular weight range would have a standard deviation of ∼2.7 Da and, therefore, it is difficult to resolve the isotopic structure (both fine structure and aggregated structure).

Also CD-MS technology is able to analyze molecules as heavy as P22 pro-capsid [1] with molecular weight above 20 MDa. The viral capsids are good examples to illustrate the usefulness of our methodology, as these particles, consisting of building blocks called protomers, have atomic composition with proportions similar to those of averagine [16]. Hence, the linear model build for peptides can be used to estimate the variance of the most abundant peak of capsid fine isotope distribution.

For illustrative purpose, consider the HIV1 capsid (∼34 MDa). The structure of the particle has recently been resolved by cryo-electron tomography at sub-nanometer resolution [17]. Although with current MS technologies the isotope pattern of HIV1 capsid particle is still not attainable, the MS analysis of intact particles is perfectly feasible. Therefore, especially for researchers who strive for ever higher mass resolution, it is important to know that the standard deviation for fine isotope structure distribution of the most abundant peak (containing about 0.25% of the full isotopic distribution and shifted by 22,018 from monoisotopic one) is ∼0.32 Da, which implies significant overlap between consecutive aggregated isotope variants that will distort the isotope profile. See further discussion in Supplementary Material on the issue of complete overlap of the fine structure envelope between consecutive peaks.

### How Far from the Normality is the Fine Isotopic Distribution?

Having computed mean, variance, entropy, spread, and size of the fine isotopic distribution of an aggregated isotopic variant, we investigate if the fine isotopic distribution can be approximated by a normal distribution. We focus here on the fine structure of the most abundant peak of the tested poly-averagines [16] (Supplementary Table 2), which can be considered as approximations of several peptides and protomers, which are building blocks of viral capsids. It should be noted that for heavier molecules, the theoretical probabilities of the most abundant isotopic variants decrease, e.g., for molecule *C*_19754_*H*_31033_*N*_5431_*O*_5909_*S*_167_ its most abundant aggregated variant with 277 additional neutrons has probability of 0.023. Of course, the fine peaks within this cluster are smaller by several orders of magnitude. For example the fine peak composed of 213 atoms of ^13^*C*, 3 atoms of ^2^*H*, 21 atoms of ^15^ *N*, 2 atoms of ^17^*O*, 12 atoms of ^18^*O*, 1 atom of ^33^*S*, and 7 atoms of ^34^*S* (other atoms in monoisotopic variants) has, according to the multinomial distribution (see [9]) a probability of approximately 8 × 10^–7^ which is relatively high within this aggregated variant. As a consequence, a huge number of ions should be analyzed to have a reasonable chance to observe these fine peaks.

To assess the difference between the fine structure distribution of the most abundant isotopic peak, say *P* and the normal distribution, say *Q*, we calculate the relative entropy (also known as the Kullback–Leibler divergence):

$$ {D}_{KL}\left(P\left\Vert Q\right.\right) = {\displaystyle \sum_i \ln}\left(\frac{P(i)}{Q(i)}\right)\;P(i). $$

The relative entropy, closely related to the information theoretic entropy *H*(*P*) discussed in “Information theory entropy of the fine structure of an aggregated isotopic variant,” measures the loss of information when the model distribution *Q* is used to approximate real distribution *P*. The notion of cross-entropy between *P* and *Q*, i.e., *H*(*P*, *Q*), ties together the information theory entropy and the Kullback–Leibler divergence measure:

$$ H\left(P,Q\right) = H(P) + {D}_{KL}\left(P\left\Vert Q\right.\right). $$

The standard definition of relative entropy assumes that both distributions are of the same type (i.e., discrete or continuous). As a consequence of this assumption, we discretized the normal distribution *Q*, because the fine isotopic distribution obtained with mercury (cf. Supplementary Figure 6) is discrete. The mean and variance for the normal distribution were calculated using moment generating functions as described in the Methods section.

**Figure 4:**
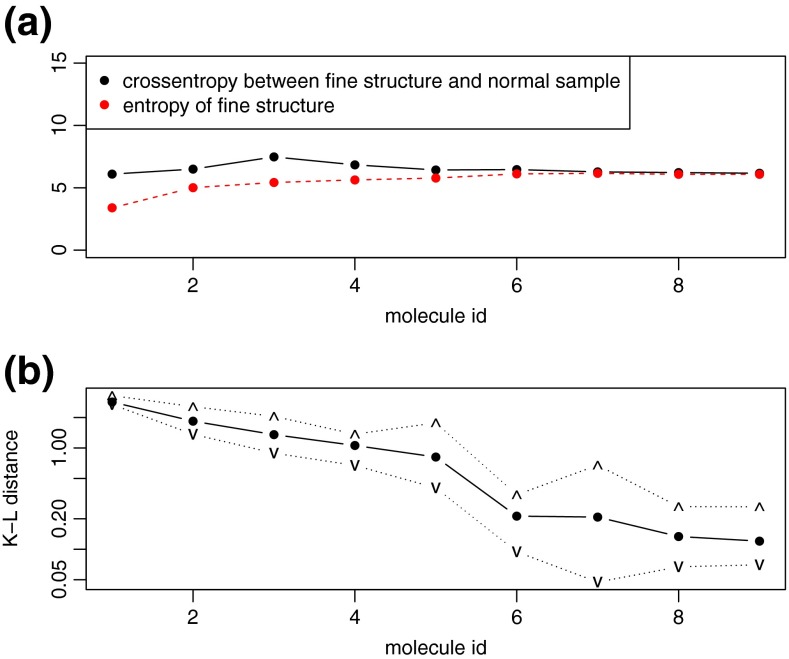
**(a)** Entropy of fine structure, and cross-entropy between fine structure of nine averagines and normal distribution; dots represent average over 10 runs; **(b)** Kullback–Leibler distance between fine structure and normal distribution (note the logarithmic y-axis); dots, ‘∧’, and ‘∨’ represent average, maximum and minimum over 10 runs, respectively. We used fine structures generated by mercury and corresponding normal distribution with mean and SD calculated using generating functions. To calculate these measures, we discretized both samples and placed them into 1,000 bins. To avoid infinite values, we added single pseudo-counts to each bin

Figure 4 illustrates the asymptotic behavior of relative entropy and cross-entropy for averagines of increasing size. The cross-entropy between *P* and *Q* tends to be similar to the entropy of distribution *P* (Figure 4a), whereas the relative entropy becomes small (Figure 4b). Similar behavior can be seen for the QQ-plot (Supplementary Figure 7). Moreover, we performed the Kolmogorov–Smirnov normality test, the Hartigans’ dip test for unimodality [18], and tests for skewness and kurtosis (see Supplementary Table 2).

As the molecular weight increases, the isotopic fine structure becomes more complex (i.e., the number of fine isotopic peaks increases). Due to this complexity, it is difficult to calculate the exact fine structure of the isotopic variants with high number of the additional neutrons (please, note that mercury does not produce exact fine peaks, but interpolates the fine structure on the dense grid). Comparing the relative abundance of the (interpolated) fine isotopic distribution with the normal distribution identified several important factors. First, as could be expected, as the molecular weight of the isotopic variant increases, the overall envelope of the isotopic fine structure cluster becomes wider. Moreover, as the molecular weight increases, the number of fine structure peaks within a certain mass interval becomes so large that the fine structure peaks become unresolved, even at a FWHM of 4.412 × 10^–4^ Da. Finally, as the molecular weight increases, the isotopic fine structure cluster becomes more symmetrical and the visual aspect of the isotopic fine structure cluster appears to become more Gaussian. Based on this last observation, we claim that at least for the most abundant peaks of large poly-averagines, the normal distribution is a good approximation of the fine isotopic distribution. Although formal testing of the normality assumption is advisable, we point out that statistical tests for normality generally reject the normality hypothesis for distributions with a large number of data values (N) attributable to the statistical power, which increases if N is large.

### How Would Thermorelativistic Effects Influence Isotope Resolution?

It turns out that parameters characterizing the fine isotopic distributions estimated using our methodology give insight into potential limitations of the concept of the isotopic resolution (i.e., the limits for distinguishing individual peaks or the distinguishing of fine structure components). Moreover, in this section we discuss the phenomena related to thermorelativistic effect of mass uncertainty, which also leads to the fundamental limit that can only be mitigated by cooling the ions.

The relativistic limit to resolution in mass spectrometry arises from thermal energy fluctuations. One can investigate this effect using fluctuation theory from thermal physics. In the present context, we address the question of whether this may limit the ability to resolve the isotopic fine structure.

Using the abovementioned methods for an averagine protein ion with a chemical formula of *C*_9877_*H*_15517_*N*_2715_*O*_2955_*S*_83_ the energy uncertainty is equivalent to a mass uncertainty of ∆*m* = 9.98 × 10^–36^ kg or ∆*m* = 6.00 × 10^–9^ Da; (detailed calculations can be found in Supplementary Material). Thus, any two fine structure components that are separated by less than 6.00 × 10^–9^ Da will not be resolved when the masses of an ensemble of ions are measured, even with a hypothetical infinite resolution mass spectrometer. This limit is a fundamental limit that can only be mitigated by cooling the ions.

Ion cooling has been shown to improve both resolution and signal levels [19]. Various methods of ion cooling have been employed, and extensive discussions being given in references [20, 21]. Most methods involve translational cooling. However, translational cooling can only lead to cooling of the internal modes of motion if there is a coupling mechanism between translational energy and internal energy of the ions.

With regard to the thermorelativistic effect, cooling of the internal modes of motion is of primary importance. It is beyond the scope of this paper to discuss these in detail, but cooling ions while within the mass analyzer is possible for some trapping-type mass analyzers, such as ion cyclotron resonance, and cooling prior to injecting ions into the mass analyzer is required for certain other mass analyzers, such as time-of-flight. There are two ways that internal motion can be cooled while ions are in the mass analyzer. One is via collisions with background gas (vibrational to translational energy exchange), and the second is via radiative cooling (primarily via infrared emission by the ions and absorption by the walls of the instrument). One might expect neither process to be very efficient, and thermodynamics requires that in both cases the body receiving the energy must be colder than the ions being cooled. For instruments that would require cooling prior to injection of ions into the analyzer, cooling of internal modes of motion via entrainment of ions in a supersonic beam is a possibility. Also, related to cooling, one would need to avoid anything that would reverse the cooling process. These undesirable processes would potentially include high energy collisions and infrared energy transfer from the walls of the apparatus to the ions. In general, cooling of ions sufficiently to overcome the thermorelativistic effect is likely to be a very considerable technical challenge. We also note that based on the used equations, the thermorelativistic mass uncertainty, ∆*m*, will be proportional to the square root of the molecular weight of the protein.

Next, we estimate the average separation between fine structure peaks in the most abundant aggregated variant of this molecule (cf. Supplementary Material for calculations). Based on an extremely conservative estimate, an average peak spacing occurs to be of 7.69 × 10^–11^ Da per fine structure peak. This is almost two orders of magnitude smaller than the estimated thermorelativistic mass spread (6.00 × 10^–9^ Da, as calculated earlier). We can therefore expect that a large number of the fine structure peaks are unresolvable because of the thermorelativistic effect.

An alternative calculation that is still conservative, but less conservative than the one just concluded, estimates a peak spacing of 1.88 × 10^–11^ Da, which is over two orders of magnitude less than the thermorelativistic peak width. This is based on an estimate of peak width of ± two standard deviations from the mean of the isotopic fine structure cluster (*σ* = 0.026 Da).

Interpreting this result requires taking several aspects into consideration. This result does not take into account the relative abundance of the fine structure peaks. It seems likely that the abundance of most of the fine structure peaks will be very low, leaving much of the abundance to be concentrated into fewer peaks of higher abundance. In that case, it may be possible for the most abundant features to be resolved, or at least as a distinct feature rising above the background of less abundant and unresolved isotopic fine structure peaks, even though most of the fine structure features would be unresolved. This is a topic best reserved for future investigation. Other estimates and assumptions are discussed in Supplementary Material.

Note also that the information theory entropy gives a measure of the degree of complexity of an isotopic fine structure cluster. Although we do not propose a specific mathematical relationship in this paper, it seems clear that an increase in information theory entropy is correlated with the onset of the significance of the thermorelativistic effect in limiting resolution. This relationship is a topic for future investigation.

### General Comments and Limitations of this Study

In this section, we discuss the scope of limitations on the fine isotopic distribution measurements and the influence of other factors contributing to the limit of the fine isotopic distribution.

Notice that this paper makes an assumption that we are dealing with an ideal instrument i.e., one in which the observed spectrum corresponds to a theoretically calculable isotopic distribution convoluted with a peak shape function characteristic of the instrument but independent of other factors, such as the number of ions loaded into the instrument. It also assumes that the resolution of the instrument can be varied at will and without limitation. In addition, part of the paper assumes that one can measure an effectively infinite number of ions, which in practical terms means enough ions so that counting statistics do not limit the accuracy to which isotopic peak profiles can be determined. These assumptions can be considered as establishing a baseline to which future discussions of these issues can be referred.

Let us briefly discuss in qualitative terms the implication of the breakdown of some of these assumptions. Given the large universe of possible experimental conditions, it is not possible to give an exhaustive discussion of all possible cases, but discussion of a few may be enlightening.

#### Counting Ions

Consider first the fact that in any real experiment it is only possible to detect a finite number of ions. If we assume the ions in any given experiment are randomly distributed according to the theoretically calculated distribution, the experimentally acquired mass spectrum will contain noise due to ion counting statistics. The amount of noise can be predicted by simple statistical considerations, and the effect is to obscure features in the mass spectrum that may otherwise be apparent in the spectrum. This is discussed in more detail in an earlier section and in the Supplementary Material. In addition to the example discussed in the Supplementary Material, this issue applies generally to the problem of characterizing isotopic distributions, including but not limited to discussions of the thermorelativistic effect and nearly all other aspects of isotope studies, such as the theoretical calculations illustrated in Figure 1.

Considering this idea further and using the example discussed earlier with a molecular formula of *C*_9877_*H*_15517_*N*_2715_*O*_2955_*S*_83_, the most abundant isotopic peak would contain 2 × 10^10^ theoretical fine structure peaks (the estimated number of fine peaks with 142 neutrons more than the monoisotopic peak, composed of any number of carbons, hydrogens, nitrogens, oxygens, and at most 83 sulphur atoms). The majority of these fine structure components will contain relatively negligible abundance. For sake of discussion, let us arbitrarily assume that most of the theoretical abundance is concentrated into only 0.01% of the fine structure peaks, leaving 2 million fine structure peaks to account for most of the total abundance in the isotopic fine structure cluster. Let us further suppose that these major fine structure peaks are of roughly equal theoretical abundance. If one were to experimentally detect 200,000 ions in this isotopic fine structure cluster, each of the major fine structure components would contain, on average, 0.1 ions. In other words, only a relatively small minority of the major fine structure peaks would be populated with any ions, and the most likely occupancy of a fine structure peak that is populated is just one ion. This is clearly not enough ions to give a good experimental characterization of the fine structure pattern, even in an infinite resolution mass spectrometer. Although the correct numbers for the relative fine structure abundances used for this feasibility calculation are presently unknown, it nevertheless seems clear that this line of thought is conceptually correct in the sense that once the isotopic fine structure pattern reaches a certain level of complexity, it would become impractical to determine the isotopic fine structure cluster profile to even a relatively crude level. Ion counting statistics can therefore represent a formidable practical limit to the usefulness of high resolution mass spectrometry, even in a hypothetical mass spectrometer of infinite resolution. More detailed consideration of these effects remains a topic for future study.

#### Interactions of Ions

Consider next the fact that ions in a real mass spectrometer may undergo various non-ideal interactions. For example, ions interact with each other via the Coulomb interaction. In mass spectrometers in which ions are bunched in space and time, this can lead to perturbations of ion trajectories. This can perturb the isotopic peak profiles. Instruments that rely on ion trapping are particularly susceptible to this sort of thing. For example, in ion cyclotron resonance mass spectrometry, the phenomenon of phase- or frequency-locking has been studied by several research groups [22, 23] and it is known that this can lead to significant distortions of spectra, including the locking of ions of closely spaced frequencies into a single peak. In general, this is of particular concern when the frequency splitting of real peaks is very small, which of course makes isotopic fine structure peaks particularly susceptible to distortion. When this happens, the isotopic fine structure becomes unresolved, which represents an additional limit to resolution not covered by the earlier discussion in this paper.

#### Fourier Transform Mass Spectrometry

An additional form of distortion can occur during peak processing from Fourier transform mass spectrometers [24]. For example, the closely spaced frequency components of an isotopic fine structure cluster, when combined with apodization and Fourier transform signal processing, can result in severe distortions of the isotopic peaks, potentially including both abundance distortions and peak shape distortions relative to the expected peak shapes and abundances. In general, these distortions are most severe when the instrument is not quite able to resolve the fine structure components. This limits the usefulness of high resolution measurements if the isotopic fine structure is not fully resolved. The effect is not necessarily a small one, and based on the trends discussed earlier in the present paper, this will happen with increasing molecular weight for proteins or other compound classes with a rich isotopic fine structure.

Also worth noting is that the resolution of Fourier transform depends on the acquisition time of the transient: higher resolution requires a longer transient and, furthermore, in order to achieve full resolution it requires that the transient not decay away significantly during signal acquisition. This ultimately imposes limits, not on the analysis above, but rather on the practical utility of performing the experiments since the time for the experiment may become impractically long. Analogously, in a time-of-flight instrument the path length required to achieve high resolution may become impractically long.

#### Field Inhomogeneities

In Fourier transform ion cyclotron resonance mass spectrometers inhomogeneity of the electric and/or magnetic fields can lead to loss of resolution [25]. The magnitude of these effects depend on the specific instrumentation and operating parameters. One would need to estimate these on a case by case basis before concluding whether they would limit resolution before the principal effects discussed in this paper would become dominant. Field inhomogeneities could also affect resolution in several other mass spectrometer types, including but not limited to orbitrap and time-of-flight mass spectrometers.

#### Ion/Neutral Collisions are Another Non-Ideal Effect

In Fourier transform mass spectrometers, collisions can cause a premature decay of the transient, leading to lower resolution, which would exacerbate the problems discussed earlier in this paper. Furthermore, most other forms of mass analyzers are subject to degradation of performance because of collisions with background gas. For example, in time of flight mass spectrometry collisions may alter the flight time or even scatter ions to the extent that they do not strike the detector.

#### Resistive Signal Dampening

Similarly, if the transient length were to become long enough, the signal detection process itself would begin to limit the transient length via coupling of ion motion to the electrical resistance of the signal detection circuitry. This will cause a decay of ion motion, hence a decay of the signal and a loss of resolution. Under normal conditions, this is not likely to be a limitation, but for a protein or other molecule of high molecular weight, the isotopic fine structure may be so densely packed that it would require an excessively long transient in order to resolve, and then signal attenuation due to resistive loads could become an issue. The general strategy to estimate this effect is presented in Supplementary Material.

#### The Thermorelativistic Effect May Be Especially Difficult to Overcome

As mentioned earlier, this can only be overcome by cooling of the ions, but the ultimate limits to this technique are hard to estimate. Nevertheless, it is possible to state that cooling may ultimately be limited by the temperature of the instrument walls. Owing to radiative heat transfer between the wall and the ion, it would seem to be practically impossible to cool ions below a few degrees Kelvin, and given that the isotopic fine structure undergoes a combinatorial explosion as molecular weight increases, even extensive cooling is not likely to extend the thermorelativistic limit to much higher molecular weight than it would be at room temperature. Most of the limitations discussed in the present section imply that the calculations presented in this paper are optimistic and therefore represent ultimate limitations under ideal conditions. Under more practical conditions, the limits to isotopic resolution are likely to occur even sooner than those discussed in this paper.

Finally, it is extremely difficult to appropriately quantify the effects that limit resolution in such a theoretical study. Based on our experience, we propose the following (very rough) order for some of these effects, starting from the most limiting: FT-ICR transient length > ion-neutral collisions > ion counting statistics > ion–ion interactions > field inhomogeneities > dephasing/apodization in FT signal processing > resistive signal dampening > thermorelativistic effects.

However, it must be realized that these effects might best be thought of in terms of a multidimensional matrix rather than a simple linear series, and the ordering of the importance of the various effects could change drastically, depending on the details of the instrumentation used, the experimental conditions, the signal processing schemes used, and the specific chemical species being studied. Furthermore, the ways in which these factors interact between each other and with the isotopic structure and fine structure are likely to be very complex and therefore difficult to predict using simple concepts.

## Conclusions

In this study, we proposed methods for both modeling the fine structure distribution and investigating some limits of molecular fine structure in real experiments. To this aim, we analyzed: (1) moment generating functions for calculating the variance and information theory entropy; (2) theoretical spread and number of peaks of the center-mass within the most abundant aggregated peaks; (3) the normality of the fine structure distribution for the most abundant aggregated variants; (4) thermorelativistic effects corresponding to the high-resolution measurements.

Calculated parameters, such as the first two moments of nominal isotopic peaks, provide an estimate of the width of the peaks. Empirical relationships between the molecular weights of two classes of biopolymers (proteins and nucleic acids) were given and used to estimate the molecular weight ranges for which adjacent fine isotopic clusters begin to overlap as well as for which overlap is essentially complete.

The striking aspect of this work is that it uncovers at least two rather fundamental limits to resolution for large molecules and one relative limit that depends on the number of fine structure peaks within a certain mass interval and the available resolution of an instrument. One is when the isotopic fine structure clusters become so broad that adjacent isotope peaks (peaks with different nucleon number) overlap, either partially or fully. Once full overlap occurs, there is no practical hope of resolving the isotopic peaks, even at the 1 Da level. Even if an infinite-resolution mass spectrometer would be available, the tangling of the isotopic fine structure between adjacent isotopic peaks would make the interpretation of the mass spectra virtually impossible. The second limitation arises from the thermorelativistic effect. As discussed earlier, this is a rather fundamental limit to the usefulness of ultrahigh resolution measurements of extremely large biomolecules that can only be mitigated by cooling the ions. Despite the uncertainties and approximations in the calculations based on thermal energy fluctuations, the estimates for resolution limits can serve as a warning that thermorelativistic effects cannot be ruled out a priori, even for ions as light as a few hundred thousand Da, and due to the combinatorial explosion in the number of fine structure peaks with increasing molecular weight, the thermorelativistic effect will rapidly become more important as molecular weight increases.

With today’s technology, instrumental and operational factors will limit resolution before one reaches the thermorelativistic limit. However, when considering this limit, one must keep in mind that improvements of instrumentation cannot overcome it. Even an infinite-resolution mass spectrometer cannot overcome the thermorelativistic limit. The only way to avoid the thermorelativistic limit is by either cooling the ions prior to mass analysis or by dealing only with ions with little or no isotopic fine structure, such as carbon clusters or CsI clusters.

The relative limit referred to previously in this section is implicit in Figure 1 and relates to the number of fine structure peaks within a certain mass interval in the isotopic fine structure cluster. When this “density of states” becomes too large, the spacing between the fine structure peaks becomes too small to resolve. This limit will depend on the resolution available on a given instrument as well as the compound and peak observed, but in the example illustrated in Figure 1 (bovine serum albumin), the average spacing between the fine structure peaks would already be too fine to fully resolve, even for an instrument of resolution 300,000,000. At a resolution of 76,000,000, the isotopic fine structure is washed out nearly completely (data not shown), and at any resolution much less than ∼76,000,000, there would not even be a hint of isotopic fine structure evident in the fine structure cluster, other than an unresolved overall broadening of the peak. Clearly, even for compounds well within the molecular weight range easily accessible to current instrumentation, the resolution of isotopic fine structure would be extremely challenging for some compounds.

Moreover, we observed that for large molecules, the analyzed distributions are distorted by a great number of extremely small fine peaks (which can be indistinguishable from the noise). This problem may be handled by truncating the isotopic fine structure distribution according to an appropriately defined signal-to-noise ratio, but truncation comes at a cost of distortion of the distribution, and distortions attributable to truncation tend to increase as isotopic complexity increases, or roughly speaking as molecular weight increases.

It should be noted that although we investigate a family of poly-averagines as approximation of peptides and proteins, the methodology presented in this manuscript can be easily extended for a more general set of molecules. Moreover, besides the limits discussed in this paper, there may be also additional impassable limits to mass resolution. We do hope that further research and discussion on this topic will be inspired by our study.

### Electronic Supplementary Material

ESM 1 (PDF 461 kb)

## Appendix

### Variance of the Fine Isotopic Distribution of an Aggregated Isotopic Variant

*E*(*m*_*j*_^2^) may be written explicitly using the following formula:

9 $$ E\left({m}_j^2\right) = \frac{{\displaystyle {\sum}_k{m}_{jk}^2{p}_{jk}}}{{\displaystyle {\sum}_k{p}_{jk}}} $$

Notice that the denominator corresponds to an aggregated isotopic distribution and, therefore, can be calculated using already existing methods [9].

To calculate the numerator of Equation (9) we consider the following polynomial:

10 $$ T\left(I;v,w,x,y,z\right)={\displaystyle \sum_j{\displaystyle \sum_k{m}_{jk}^2{p}_{jk}{I}^i={\displaystyle \sum_j{q}_k^{\perp }}}}{I}^j $$

which coefficients (in standard form), i.e., *q*_*j*_^⊥^≡∑_*k*_*m*_*jk*_^2^*p*_*jk*_, are the objects of our interest^.^

Moreover, for chemical compound *Y* we define the polynomial:

11 $$ {R}_A\left(I,J,K\right)={{\displaystyle \sum_j{p}_{A,j}j}}^{m_{A,j}}\;{K}^{{}^{m_{A,j}}}{I}^j $$

We introduce also polynomials:

12 $$ {P}_A\;(I)={\displaystyle \sum_j{p}_{A,j}{m}_{A,j}{I}^j} $$

and

13 $$ {P}_A^{\ast}\;(I)={\displaystyle \sum_j{p}_{A,j}{m}_{A,j}^2{I}^j} $$

Finally, let us consider the polynomial:

14 $$ \begin{array}{ll}{Q}^{\perp}\;\left(I,\ J,K;\ v,w,\ x,\ y,\ z\right)\hfill & \begin{array}{l}={R}_C{\left(I,J,K\right)}^v\times {R}_H{\left(I,\ J,K\right)}^w\times {R}_N{\left(I,\ J,K\right)}^x\times \hfill \\ {}\times {R}_O{\left(I,\ J,K\right)}^y\times {R}_S{\left(I,\ J,K\right)}^z\hfill \end{array}\hfill \end{array} $$

which can be alternatively written in its standard form:

15 $$ {Q}^{\perp}\;\left(I,\ J,K;\ v,w,\ x,\ y,\ z\right)={\displaystyle \sum_j\left({\displaystyle \sum_k{p}_{jk}{J}^{m_{jk}}{K}^{m_{jk}}}\right){I}^j} $$

Differentiating polynomial *Q*^⊥^(*I*, *J*, *K*; *v*, *w*, *x*, *y*, *z*) over *J* and *K* we obtain:

16 $$ \frac{\partial^2}{\partial J\partial K}{Q}^{\perp}\left(I,\ J,K;\ v,w,\ x,\ y,\ z\right)={\displaystyle \sum_j\left({\displaystyle \sum_k{m}_{jk}^2{p}_{jk}{J}^{m_{jk}-1}{K}^{m_{jk}-1}}\right){I}^j} $$

and by setting *J* = *K* = 1 we have the following identity:

17 $$ \frac{\partial^2}{\partial J\partial K}{Q}^{\perp}\left(I,\ J,K;\ v,w,\ x,\ y,\ z\right)\;\left|{}_{J=K=1}=T\right.\left(I;\ v,w,\ x,\ y,\ z\right) $$

On the other hand, we have:

18

where ⋆ states for a sum of 24 other polynomial products. Hence the problem of calculating coefficients *q*_*j*_^⊥^ is reduced to the calculation of polynomials that appear in Equation (18). Finally, the variance of centered masses may now be obtained from Equations (3) and (9).

To compute the variance of the center mass for a given aggregated isotopic variant, we should calculate sums of products of polynomials. Here, we apply two approaches: FFT - using explicitly fast Fourier transform; fft function in R, and a library for multiplying polynomials; PolynomF library in R [26] (which is more accurate than FFT, but also significantly slower).

To calculate the total variance of the isotopic distribution that includes the fine structure, we start from its definition:

19 $$ \begin{array}{ll}{\mathrm{Var}}_{tot}\hfill & ={\displaystyle \sum_{j,k}{p}_{jk}{m}_{jk}^2-{\overline{m}}^2={\displaystyle \sum_{j,k}{p}_{jk}{m}_{jk}^2-{\displaystyle \sum_{j,k}{p}_{jk}{m}_j^2+{\displaystyle \sum_{j,k}{p}_{jk}{m}_j^2-{\overline{m}}^2}}}}\hfill \\ {}\hfill & ={\displaystyle \sum_{j,k}{p}_{jk}{m}_{jk}^2-{\displaystyle \sum_j{q}_j{m}_j^2+{\displaystyle \sum_{j,k}{p}_{jk}{m}_j^2-{\overline{m}}^2}}}\hfill \\ {}\hfill & ={\displaystyle \sum_{j,k}{q}_j\frac{p_{jk}}{q_j}{m}_{jk}^2-{\displaystyle \sum_j{q}_j{m}_j^2+{\displaystyle \sum_{j,k}{p}_{jk}{m}_j^2-{\overline{m}}^2}}}\hfill \\ {}\hfill & ={\displaystyle \sum_j{q}_j\left({\displaystyle \sum_k\frac{p_{jk}}{q_j}{m}_{jk}^2-{m}_j^2}\right)+{\displaystyle \sum_{j,k}{p}_{jk}{m}_j^2-{\overline{m}}^2}}\hfill \\ {}\hfill & ={\displaystyle \sum_j{q}_j\mathrm{V}\mathrm{a}\mathrm{r}\;\left({m}_j\right)+{\displaystyle \sum_j{q}_j{m}_j^2-{\overline{m}}^2={\displaystyle \sum_j{q}_j\mathrm{V}\mathrm{a}\mathrm{r}\;\left({m}_j\right)+\mathrm{V}\mathrm{a}\mathrm{r}(m)}}}\hfill \end{array} $$

where we use that *q*_*j*_ = ∑_*k*_*p*_*jk*_ and the average of the total isotopic distribution is denoted by $\overline{m}$.

### Information Theory Entropy of Fine Structure for Aggregated Variant

The information theory entropy for *j*-th isotopic aggregated variant (considered as a local probability distribution) can be defined as:

20 $$ H(j)=-{\displaystyle \sum_k\frac{p_{jk}}{{\displaystyle {\sum}_k{p}_{jk}}} \log}\;\left(\frac{p_{jk}}{{\displaystyle {\sum}_k{p}_{jk}}}\right) $$

After applying the following transformation:

21 $$ {\displaystyle \sum_k{p}_{jk} \log}\frac{1}{{\displaystyle {\sum}_k{p}_{jk}}}={\displaystyle \sum_k{p}_{jk}\left\{- \log \left({\displaystyle \sum_k{p}_{jk}}\right)\right\}}=\left({\displaystyle \sum_k{p}_{jk}}\right)\; \log\;\left({\displaystyle \sum_k{p}_{jk}}\right) $$

to the previous formula, we finally obtain the Equation (5).

The formula for the information theory entropy *H* of the full isotopic distribution (fine structure included) can be derived starting from its definition

22 $$ \begin{array}{ll}H\hfill & =-{\displaystyle \sum_{j,k}{p}_{jk} \log \left({p}_{jk}\right)}=-{\displaystyle \sum_j{\displaystyle \sum_k{q}_j\frac{p_{jk}}{q_j} \log \left({p}_{jk}\right)=-}}{\displaystyle \sum_j{q}_j{\displaystyle \sum_k\frac{p_{jk}}{q_j} \log \left({p}_{jk}\right)}}\hfill \\ {}\hfill & =-{\displaystyle \sum_j{q}_j}{\displaystyle \sum_k\frac{p_{jk}}{q_j}\left( \log \left({p}_{jk}\right)- \log \left({q}_j\right)+ \log \left({q}_j\right)\right)}\hfill \\ {}\hfill & =-{\displaystyle \sum_j{q}_j}{\displaystyle \sum_k\frac{p_{jk}}{q_j}\left( \log \left({p}_{jk}\right)- \log \left({q}_j\right)\right)-{\displaystyle \sum_j{q}_j}{\displaystyle \sum_k\frac{p_{jk}}{q_j} \log \left({q}_j\right)}}\hfill \\ {}\hfill & =-{\displaystyle \sum_j{q}_j{\displaystyle \sum_k\frac{p_{jk}}{q_j}\left( \log \frac{p_{jk}}{q_j}\right)-}}{\displaystyle \sum_j{q}_j \log \left({q}_j\right){\displaystyle \sum_k\frac{p_{jk}}{q_j}}}\hfill \\ {}\hfill & ={\displaystyle \sum_j{q}_jH(j)}-{\displaystyle \sum_j{q}_j \log \left({q}_j\right)\frac{{\displaystyle {\sum}_k{p}_{jk}}}{q_j}=}{\displaystyle \sum_j{q}_jH(j)-{\displaystyle \sum_j{q}_j \log \left({q}_j\right)\frac{q_j}{q_j}}}\hfill \\ {}\hfill & ={\displaystyle \sum_j{q}_jH(j)}-{\displaystyle \sum_j{q}_j \log \left({q}_j\right)}\hfill \end{array} $$

where we use that *q*_*j*_ = ∑_*k*_*p*_*jk*_.

### Validation of the Proposed Methods for the Selected Molecules

Aiming to validate our algebraic approach to calculate theoretic variance of the fine structures, we calculated several distributions for center mass variance for selected biomolecules using polynomial method and FFT (Supplementary Figure 3A–C). Both methods are comparably accurate; however, FFT is computationally more efficient. Moreover, to validate our results, we ran isoDalton [8] and calculated exact masses for bovine serum albumin or BSA with the 100,000 and 1,000,000 most probable exact masses. We observed (Supplementary Figure 3D) that when the number of simulated peaks is big enough, the variances obtained from algebraic formulae are consistent with those simulated by isoDalton. However, selecting a limited number of most abundant peaks, the “true” shape of the fine isotopic distribution cannot be obtained. Our theoretical calculations can be used as a tool to determine the appropriate parameters (such as the number of peaks) for algorithms such as isoDalton, especially when one is interested in a particular aggregated isotopic variant.

The calculations of information theory entropy are presented in Supplementary Figure 2, which shows the entropy for isotopic clusters of selected biomolecules. As expected, for the first aggregated isotopic variants, the entropy increases, as the fine isotopic distribution becomes more and more complex.

### Brief Overview of Ultrahigh Resolution Using the Mercury Program

The ultrahigh resolution fast Fourier transform (FFT) method [7] is included as an option in the mercury computer program. Briefly, mercury relies on the convolution theorem to transform a convolution problem (the calculation of molecular isotopic distributions from the atomic isotopic distributions) into a problem that can be solved using the FFT. The ultrahigh resolution option does a zoom calculation over a user selectable limited mass region and uses digital filtering to prevent aliasing from nearby isotopic peaks. The result is an ultrahigh resolution simulated profile-mode spectrum of an isotopic cluster over a limited mass range, typically 0.2 Da.

## References

[1] Keifer DZ, Pierson EE, Hogan JA, Bedwell GJ, Prevelige PE, Jarrold MF (2014). Charge detection mass spectrometry of bacteriophage P22 procapsid distributions above 20 MDa. Rapid Commun. Mass Spectrom..

[2] Wang X, Chen H, Lee J, Reilly PTA (2012). Increasing the trapping mass range to *m/z* = 10^9^—a major step toward high resolution mass analysis of intact RNA, DNA, and viruses. Int. J. Mass Spectrom..

[3] Lin H-C, Lin J-L, Lin H-H, Tsai S-W, Yu AL, Chen RLC, Chen C-H (2012). High-speed mass measurement of nanoparticle and virus. Anal. Chem..

[4] Havlicek V, Lemr K, Schug KA (2013). Current trends in microbial diagnostics based on mass spectrometry. Anal. Chem..

[5] Lössl P, Snijder J, Heck AJ (2014). Boundaries of mass resolution in native mass spectrometry. J. Am. Soc. Mass Spectrom..

[6] Rockwood AL, Van Orden SL, Smith RD (1995). Rapid calculation of isotope distributions. Anal. Chem..

[7] Rockwood AL, Van Orden SL, Smith RD (1996). Ultrahigh resolution isotope distribution calculations. Rapid Commun. Mass Spectrom..

[8] Snider RK (2007). Efficient calculation of exact mass isotopic distributions. J. Am. Soc. Mass Spectrom..

[9] Valkenborg D, Mertens I, Lemière F, Witters E, Burzykowski T (2012). The isotopic distribution conundrum. Mass Spectrom. Rev..

[10] Claesen J, Dittwald P, Burzykowski T, Valkenborg D (2012). An efficient method to calculate the aggregated isotopic distribution and exact center masses. J. Am. Soc. Mass Spectrom..

[11] Dittwald P, Claesen J, Burzykowski T, Valkenborg D, Gambin A (2013). BRAIN: a universal tool for high-throughput calculations of the isotopic distribution for mass spectrometry. Anal. Chem..

[12] Fernandez-de-Cossio Diaz J, Fernandez-de-Cossio J (2012). Computation of isotopic peak center mass distribution by Fourier transform. Anal. Chem..

[13] Böcker, S. Lipták, Z.: Efficient mass decomposition. Proceedings of the 2005 ACM Symposium on Applied computing, SAC ’05, pp. 151–157, New York, NY (2005)

[14] The UniProt Consortium: Reorganizing the protein space at the universal protein resource (UniProt). Nucleic Acids Res. **40**, D71–D75 (2012) 10.1093/nar/gkr981 PMC3245120 22102590

[15] Chen R, Cheng X, Mitchell DW, Hofstadler SA, Wu Q, Rockwood AL, Sherman MG, Smith RD (1995). Trapping, detection, and mass determination of coliphage T4 DNA ions by electrospray ionization Fourier transform ion cyclotron resonance mass spectrometry. Anal. Chem..

[16] Senko MW, Beu SC, McLafferty FW (1995). Determination of monoisotopic masses and ion populations for large biomolecules from resolved isotopic distributions. J. Am. Soc. Mass Spectrom..

[17] Schur FK, Hagen WJ, Rumlova M, Ruml T, Muller B, Krausslich H, Briggs JA (2015). Structure of the immature HIV-1 capsid in intact virus particles at 8.8 Å resolution. Nature.

[18] Hartigan JA, Hartigan PM (1985). The dip test of unimodality. Ann. Stat..

[19] Mitchell DW, Smith RD (1995). Cyclotron motion of two Coulombically interacting ion clouds with implications to Fourier-transform ion cyclotron resonance mass spectrometry. Phys. Rev. E.

[20] Gorshkov MV, Masselon CD, Anderson GA, Udseth HR, Harkewicz R, Smith RD (2001). A dynamic ion cooling technique for FTICR mass spectrometry. J. Am. Soc. Mass Spectrom..

[21] Itano WM, Bergquist JC, Bollinger JJ, Wineland DJ (1995). Cooling methods in ion traps. Phys. Scr..

[22] Peurrung AJ, Kouzes RT (1995). Analysis of space-charge effects in cyclotron resonance mass spectrometry as coupled gyrator phenomena. Int. J. Mass Spectrom. Ion Processes.

[23] Boldin IA, Nikolaev EN (2009). Theory of peak coalescence in Fourier transform ion cyclotron resonance mass spectrometry. Rapid Commun. Mass Spectrom..

[24] Rockwood AL, Erve JC (2014). Mass spectral peak distortion due to Fourier transform signal processing. J. Am. Soc. Mass Spectrom..

[25] Nikolaev EN, Vladimirov GN, Jertz R, Baykut G (2013). From supercomputer modeling to highest mass resolution in FT-ICR. Mass Spectrom. (Tokyo).

[26] Venables, B.: PolynomF: polynomials in R, R package. Available at http://CRAN.R-project.org/package-PolynomF.

