# Supplementary material for: On the Fine Isotopic Distribution and Limits to Resolution in Mass Spectrometry
Source: J Am Soc Mass Spectrom. 2015 Aug 12;26(10):1732–45. doi: 10.1007/s13361-015-1180-4 (PMC4565875; doi:10.1007/s13361-015-1180-4)
Supplement: Supplementary file 1 — (PDF 461 kb) [file 13361_2015_1180_MOESM1_ESM.pdf]

Supplementary materials to the manuscript entitled: On the fine isotopic distribution and limits to resolution in mass spectrometry

Piotr Dittwald[a,g], Dirk Valkenborg[b,c,d], Jürgen Claesen[d], Alan L. Rockwood[e,f,h], Anna Gambin[a,h]

[a] *Institute of Informatics, University of Warsaw, Warsaw, Poland*
[b] *Applied Bio & molecular Systems, VITO, Mol, Belgium*
[c] *Center for Proteomics, Antwerp, Belgium*
[d] *Interuniversity Institute for Biostatistics and Statistical Bioinformatics, Hasselt University, Diepenbeek, Belgium*
[e] *Dept. of Pathology, University of Utah School of Medicine*
[f] *ARUP Laboratories*
[g] *previous affiliation: College of Inter-faculty Individual Studies in Mathematics and Natural Sciences, University of Warsaw, Poland*
[h] *corresponding authors: aniag@mimuw.edu.pl and alan.rockwood@aruplab.com*

When do isotopic fine structures overlap? – analysis on the theoretical limits

The estimation when two subsequent fine structures can overlap is based on the following assumptions:

- the substantial ($\sim 31.8\%$) part of the normal distribution is located outside the interval $\bar{m} \pm \sigma$, where $\bar{m}$ and σ are its mean and standard deviation, respectively;
- the isotopic fine structure cluster tends to be normal (this condition is discussed in the main paper);
- from Eq. (8) in the main text check when $\sqrt{\mathrm{Var}(m_a)}$ reaches 0.5 Da – in such case the two consecutive distributions of mass fine structures may overlap;
- this is applied for the most abundant variant (the standard deviation of the preceding isotope aggregated variant center-mass is assumed to be very similar).

Extending the line of thought from Section 3.2, we are also interested in the approximate size of the molecule for which the overlap of the fine structure envelope between consecutive peaks becomes complete. We consider two consecutive peaks to be completely overlapping if the standard deviation of one peak is at least 1 Da. From Eq. (8) in the main text this is estimated to occur at ca. 325 MDa.

Supplementary Figure 3B illustrates the degree of overlap when the standard deviation of several peaks is 1 Da. As a result of the overlap, one peak (in black) will be shown. This composite peak is very smooth, and because of this, it is impossible to find out if this peak is a superposition of separate peaks.

In this curve, the mass axis, symbolized by the variable x, has been shifted by subtracting the mass of the most abundant peak. The central part of this composite peak can be seen as a reasonable representation of the overlap of the peaks near the most abundant peak in the distribution. Thus, under these conditions the concept of "isotopic resolution" is essentially meaningless. Therefore, there is little use for isotopic resolution (i.e. the ability to resolve peaks spaced by approximately 1 Da) mass spectrometry for extremely large protein molecules (e.g. above $\sim$ 325 MDa).

Continuing with the issue of partially resolved peaks, in a certain sense Figure 3A represents a best case. A real mass spectrum contains noise, and a significant source of noise in many cases arises from the quantized nature of ion abundance statistics. Thus, rather than being a perfectly smooth profile as illustrated in Figure 3A, a real mass spectrum will be subject to counting noise due to the randomness of detection of a finite number of ions. This counting noise may mask the structure that might be evident in a perfect noiseless signal.

For illustrative purposes, let us consider a protein with a molecular weight of 60 MDa. Each isotopic fine structure cluster (i.e. each aggregated isotopic variant) near the most abundant aggregated isotope peak, if expressed as a probability distribution, will have a standard deviation of 0.43 Da (cf. Eq. (8)). This matches the standard deviation in the widths of the nominal isotope peaks in Figure 3A.

If one performs a Monte Carlo sampling over the full isotopic distribution, using an expectation value of one million total ions, then the expectation value for the most abundant nominal isotope peak is 1921.4 counts. Ion counting follows Poisson statistics, so the standard deviation in the number of counts is $\sqrt{1921.4}$. The nearby peaks will have essentially the same expec-

tation value and standard deviation. Figure 3C gives an example of a Monte Carlo simulation of the nine peaks in Figure 3A, using the parameters just described.

The results have been binned together using a step size of 0.1 Da. This would correspond to a mass spectrometer that samples every 0.1 Da. A striking feature of this simulation is that the incipient isotopic resolution evident in Figure 3A is completely obscured by counting noise in the Monte Carlo simulation.

Using a mass spectrometer with a smaller step size, which would be required to support higher resolution, does not help. Figure 3D shows the same simulation with a step size (grid spacing) of 0.01. The isotopic structure is still masked by counting noise. The only way to avoid this problem would be to detect more ions. Unfortunately, signal/noise improves as a weak (square root) function of the number of counts.

How would thermorelativistic effects influence isotope resolution? – detailed calculations and discussion

From fluctuation theory there is an uncertainty, ΔE, in the thermal energy contained in a molecule or ion. For the purposes of this discussion we do not distinguish between and an ion and a molecule, and we may therefore use the terms interchangeably. The RMS energy uncertainty is given by:

$$\Delta E = \sqrt{kT^2 C_v} \tag{1}$$

where k is Boltzmann's constant, T is absolute temperature, and C_v is heat capacity [1, 2, 3].

One can estimate the heat capacity using the equipartition theorem. By analogy to the Debye theory of heat capacity of crystals, at room temperature we can assume that most of the vibrational degrees of freedom of a large protein molecule are low frequency modes and can therefore be considered to be fully thermally active. Each oscillator will contribute to the heat capacity, and there are three oscillators per atom. If the molecule contains vibrationally active atoms the equipartition theorem [3] predicts a heat capacity of $C_v = 3kN$. Since we are dealing with large ions we ignore the fact that there are three translational and three rotational degrees of freedom, which would effectively reduce by a count of six. After some substitutions and algebra we have:

$$\Delta E = \sqrt{3N}kT \tag{2}$$

Consider an averagine molecule with chemical formula $C_{9877}H_{15517}N_{2715}O_{2955}S_{83}$. In counting atoms to generate a value for N we exclude hydrogen atoms, because they are associated with high frequency modes which, generally speaking, are not thermally activated at ambient temperatures. We therefore calculate by counting the number of carbon, nitrogen, oxygen, and sulfur atoms in the molecule, giving a value of $N = 15,630$.

Substituting this into the Eq. (2) and assuming $T = 300$ we estimate the thermal energy uncertainty for a single ion to be $\Delta E = 8.97 \times 10^{-19} J$. Using the relationship between mass and energy from special relativity $E = mc^2$ we calculate the energy uncertainty to be equivalent to a mass uncertainty of $\Delta m = 9.98 \times 10^{-36}$ kg or $\Delta m = 6.00 \times 10^{-9}$ Da.

Next, we estimate the average separation between fine structure peaks in the molecule. Let us consider only the most abundant isotopic peak in the distribution for the molecule under consideration here, which has a molecular weight of 222, 241.8 Da. This peak contains 142 more nucleons than the monoisotopic peak. The number of isotopic fine structure components in the most abundant isotopic peak is estimated to be 1.95×10^{10} (the number of fine peaks with 142 neutrons more than the monoisotopic peak, composed of C, H, N, O, S, but with restriction not to use more than 83 sulphur atoms). The maximal theoretical spread of the fine structure is estimated as 1.5 Da (cf. Figure 3B). We therefore very conservatively estimate the fine structure density to be:

$$\frac{1.95 \times 10^{10} \text{ fine structure peaks}}{1.5 \text{ Da}} = \frac{1.3 \times 10^{10} \text{ fine structure peaks}}{1 \text{ Da}}$$

for an average peak spacing of 7.69×10^{-11} Da per fine structure peak.

Note also that basing the peak density on maximal spread results in an extremely conservative estimate. Since most of the peaks are concentrated within a much narrower region (within one or two standard deviations of the mean mass of the isotopic fine structure cluster), the peak density for most of the cluster is much higher than that calculated here. The thermorelativistic effect calculations are based on certain estimates and assumptions, partially mentioned in the main part of the manuscript. If these are violated then it could alter the interpretation. For example, the value of N used for these calculations was based on the money changer problem discussed earlier. This predicts a monotonically increasing function with increasing neutron number (Supplementary Figure 1 illustrates this for molecules composed of C, H, N, and O). However, strictly speaking, these calculations were based on a

molecule of infinite molecular weight. For any real molecule of finite molecular weight the function must turn over and return to a value of a single fine structure peak for an isotopic peak composed of all heavy isotopes. We expect, therefore, that there are some errors in the estimate of N, and that N may be overestimated though one expects the estimate to be fairly reliable as long as N is not too great. The degree of error introduced by this assumption is a topic for future investigation.

The estimate was also based on an assumption that all modes of motion are thermally activated. However, if there are many vibrational modes of very high frequency these will not be thermally activated, and the estimated heat capacity will be too high. We have taken this at least partly into account by excluding hydrogen atoms when counting N, but it is possible that N is still overestimated. This is also a topic for future investigation.

Please, note the thermorelativistic calculation was performed using the default elemental isotopic abundance and accurate mass table used by the `mercury` program. These abundance and masses differ slightly from those used elsewhere in this paper. These differences do not materially change the results.

Resistive signal dampening - general strategy to estimate limits to resolution

A signal in a Fourier transform instrument can be modeled as an alternating charge on an resistor-capacitor (RC) circuit, where the resistor and the capacitor are in parallel. The signal consists of an image charge on the capacitor, which is given by

$$Q_{cap} = N \times q \times K \times \cos(\omega t)$$

where Q_{cap} is the capacitor charge, N represents the number of ions in the trap, q represents the number of charges on a single ion, and K is a geometrical factor, which in the case of an FT-ICR instrument includes factors such as the relative cyclotron radius relative to the size of the cell and an adjustment factor relating to the single-ended character of a signal arising from an orbiting charge compared to charging a capacitor from an external source.

The voltage on the capacitor is given by $V = \frac{Q_{cap}}{C}$, where C is the capacitance of the cell. The current in the input resistance of the pre-amp of the instrument is $I = \frac{V}{R}$, where R is the input resistance. The power dissipated

in the input resistance of the pre-amp is

$$P = I \times V = \frac{Q_{cap}^2}{R}$$

If the RC time constant is long compared to the period of oscillation of the ions in the instrument one can make the following replacement

$$\cos^2(\omega t) \approx \frac{1}{2}$$

and calculate the near-instantaneous rate of dissipation of kinetic energy of the ion packet due to the input resistance of the amplifier. This will cause the cyclotron radius to decay, leading to a loss of signal over time which reduces the achievable mass resolution. In a full simulation of this effect one would need to take into account that the loss of cyclotron radius implies that K is a time-dependent quantity rather than a constant. The magnitude of the loss of resolution from this effect is highly dependent on the parameters of any specific experiment and would need to be estimated on a case by case basis.

References

[1] M.R. Evans. Statistical physics lecture notes, University of Edinburgh, UK. `www2.ph.ed.ac.uk/~martin/sp/sp3.pdf`. Accessed: 2013-09-10.

[2] M. Plischke and B. Bergersen. *The Principles of Statistical Mechanics.* World Scientific, New Jersey, 3rd edition, 2006.

[3] R.C. Tolman. *The Principles of Statistical Mechanics.* Oxford University Press, London, 1938.

Supplementary Tables and Figures

isotope	probability	mass (Da)	average mass per additional neutron (Da)
^{12}C	0.9893	12.0	–
^{13}C	0.010700	13.0034	1.0034
^{1}H	0.999885	1.0078	–
^{2}H	0.000115	2.0141	1.0063
^{14}N	0.99632	14.0031	–
^{15}N	0.00368	15.0001	0.9970
^{16}O	0.997570	15.9949	–
^{17}O	0.000380	16.9991	1.0042
^{18}O	0.002050	17.9992	1.0021
^{32}S	0.9493	31.9721	–
^{33}S	0.0076	32.9715	0.9994
^{34}S	0.0429	33.9679	0.9979
^{36}S	0.0002	35.9671	0.99875

Supplementary Table 1: Elemental distributions (i.e. probability and corresponding mass) for stable isotopes of five considered atoms (mass values are rounded to fourth decimal digit). In addition, for heavy isotopic variants, an average mass per additional neutron is given. For example for ^{18}O the obtained value is equal to $\frac{1}{2}(m_{18O} - m_{16O})$ where m_{18O}, m_{16O} refer to masses of ^{18}O and ^{16}O, respectively. Data similar to IUPAC 1997 (see Rosman *et al. Isotopic compositions of the elements 1997.* Pure Appl. Chem. 70:217–235, 1998). Please, note these values might slightly differ within the simulations described in the article.

				p-values			
id	molecule	k	*prob*	K-S test	unimodality	skewness	kurtosis
1	$C_{247}H_{388}N_{68}O_{74}S_2$	3	0.206	0	0.0634	0	1.18e-277
2	$C_{1235}H_{1940}N_{339}O_{369}S_{10}$	17	0.0906	0	0.0156	0	7.31e-11
3	$C_{2469}H_{3879}N_{679}O_{739}S_{21}$	34	0.064	0	0.00492	0	0.0164
4	$C_{3704}H_{5819}N_{1018}O_{1108}S_{31}$	51	0.0522	0	0.00408	0	6.13e-05
5	$C_{4938}H_{7758}N_{1358}O_{1477}S_{42}$	69	0.0452	0	0.0032	0	0.282
6	$C_{9877}H_{15517}N_{2715}O_{2955}S_{83}$	138	0.032	0	0.00278	0	0.102
7	$C_{14815}H_{23275}N_{4073}O_{4432}S_{125}$	207	0.0261	0	0.00221	0	0.41
8	$C_{19754}H_{31033}N_{5431}O_{5909}S_{167}$	277	0.0226	0	0.00201	4.53e-10	0.0351
9	$C_{24692}H_{38792}N_{6788}O_{7386}S_{208}$	346	0.0202	0	0.00176	2.46e-10	0.0209

Supplementary Table 2: The analysis of fine peaks distributions for the family of averagines for $N = 40,000$ peaks in fine structure distribution generated based on `mercury` software; normality is tested by Kolmogorov-Smirnov (comparison with random sample drawn from the normal distribution with mean and variance of the tested sample of size N); unimodality by Hartigan's DIP test; parameter k indicates the number of neutrons in the most abundant aggregated peak, for which the fine structure is investigated, column *prob* presents the probability of this (aggregated) peak.

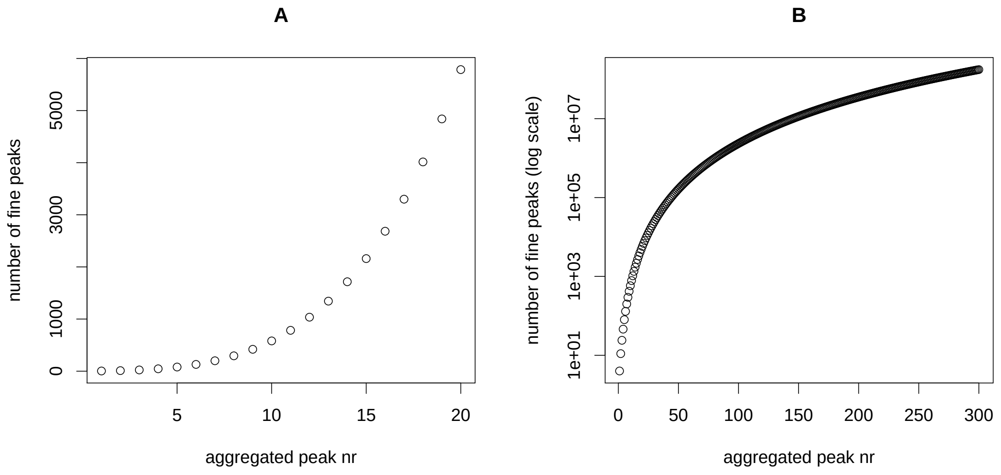

Supplementary Figure 1: Theoretical number of fine peaks for each aggregated variant (Panel A presents the relation for smaller numbers of additional neutrons in comparison to Panel B). Please, note the logarithmic scale on Panel B y-axis. For this figure we used a `decomp` tool and investigated only stable isotopes from four elements (C, H, N, O). However, if sulfur were included the number of fine peaks would be greater.

A B

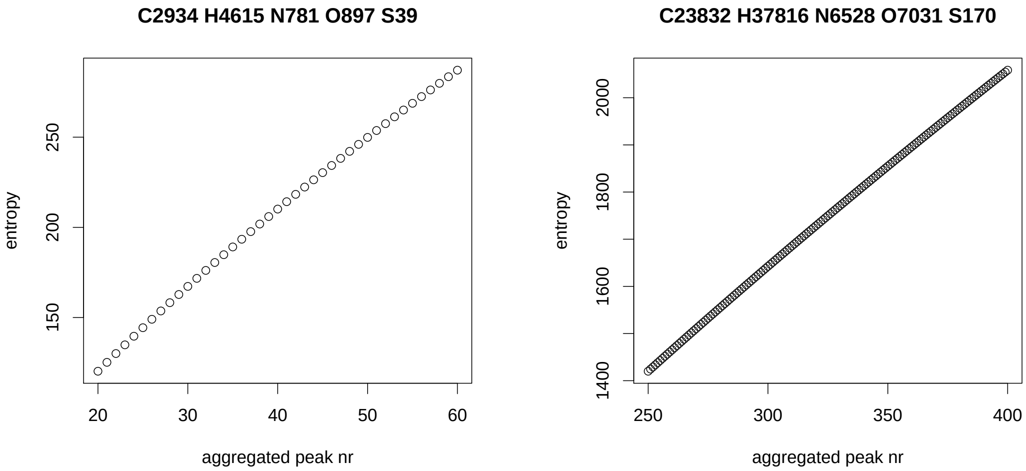

Supplementary Figure 2: Information theory entropies for aggregated variants for (A) Bovine serum albumin - most abundant aggregated variant has number 42, and (B) Human dynein heavy chain - most abundant aggregated variant has number 330. The results are obtained by simulations based on the generating function approach introduced in Methods and using FFT implementation.

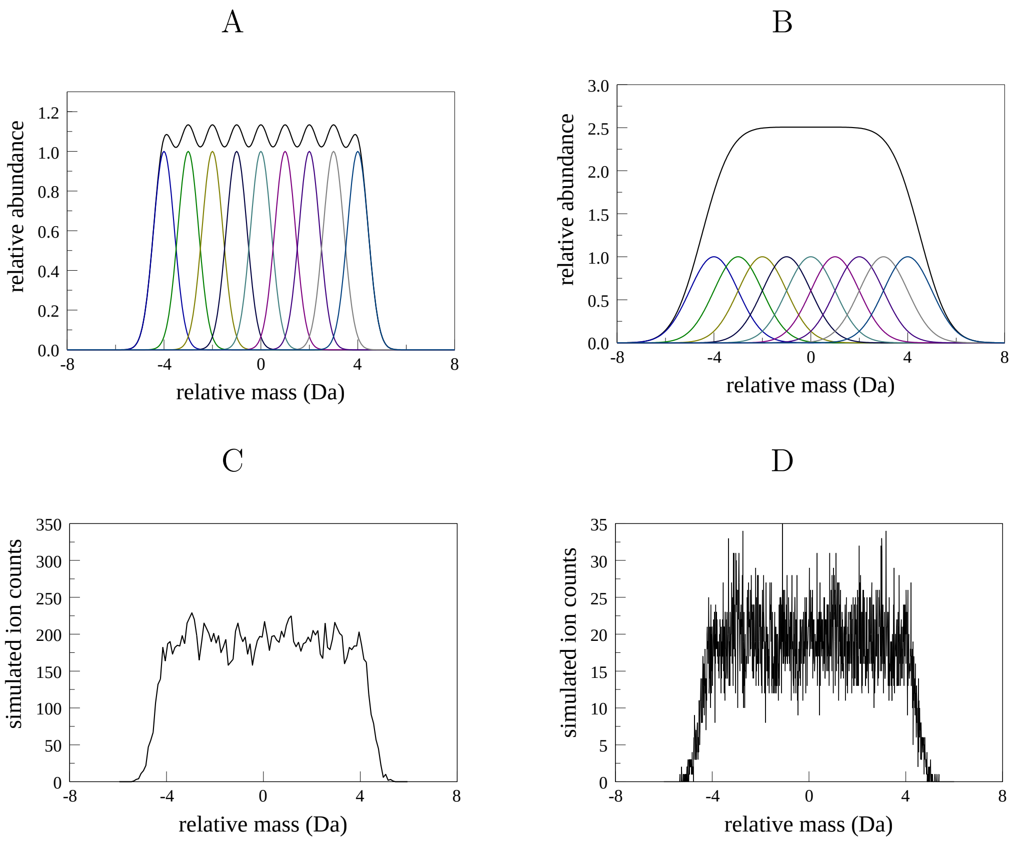

Supplementary Figure 3: If the peaks become broad enough then the overlap is not just partial (as situation presented in Panel A), but the peaks coalesce to the point where nominal isotopic peaks are not even discernible. For example, if the variance is 0.184 (i.e. a standard deviation of 0.43) then the the superposition of a peak, together with the five adjacent higher-mass peaks and the five adjacent lower-mass peaks would look like those in panel A. If the variance is 1, then adjacent isotopic peaks would look like those in Panel B. This would represent a kind of fundamental, or near-fundamental limitation on the usefulness of high resolution. Once the adjacent nominal isotopic peaks become overlapped then there isn't much point in going to higher resolution. Panels C-D present Monte Carlo simulations of overlapping peaks with FWHM of 0.43 Da (parameters for the simulations are explained in the text). Panel C corresponds to a grid spacing of 0.1 Da, and Panel D corresponds to a grid spacing of 0.01 Da. Note that the masses, depicted on the x-axis, are the differences between the peak mass and the most abundant peak mass.

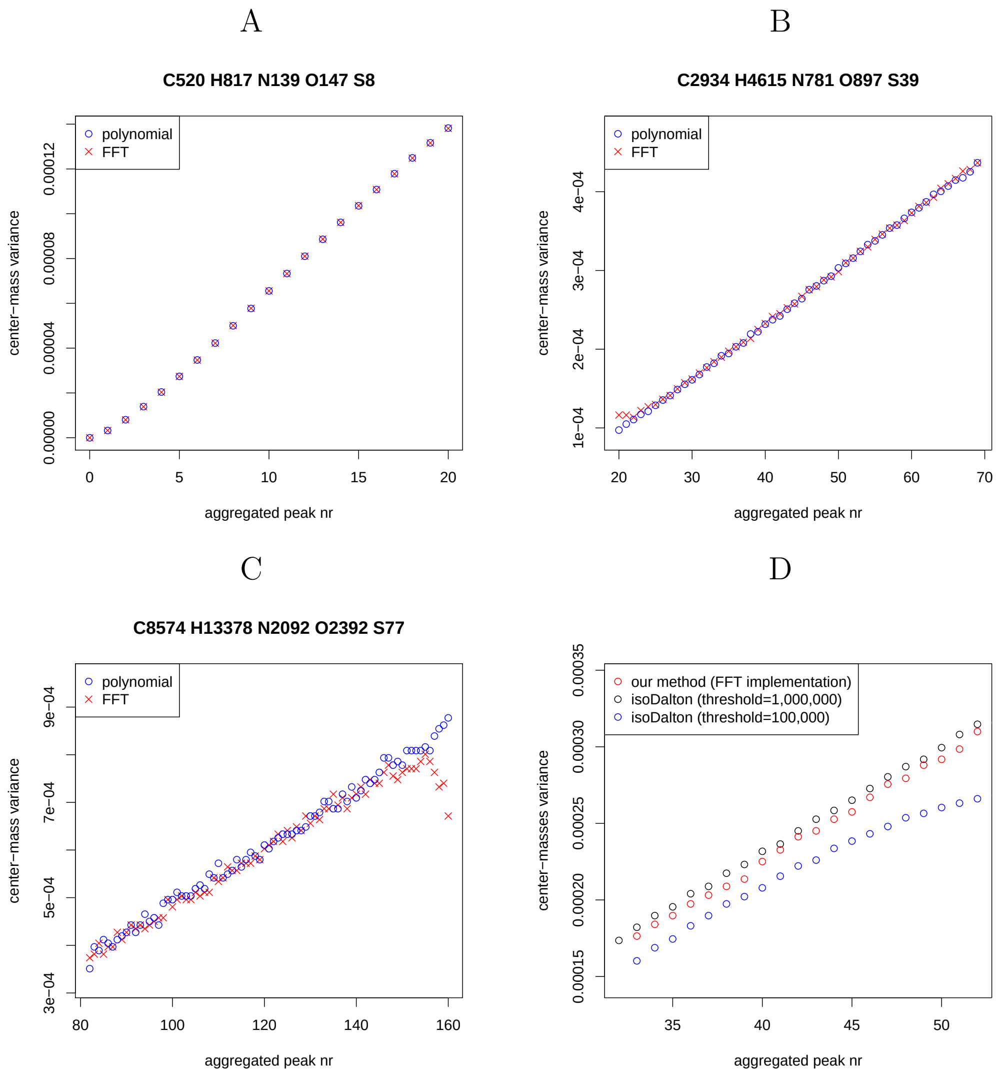

Supplementary Figure 4: The center-mass variance for most abundant peaks for following biomolecules using both polynomial (blue) and FFT (red) implementation: (A) Human insulin ($C_{520}H_{817}N_{139}O_{147}S_{8}$), (B) Bovine serum albumin ($C_{2934}H_{4615}N_{781}O_{897}S_{39}$), (C) Renal isoform, subunit Human ATP binding cassette protein ($C_{8574}H_{13378}N_{2092}O_{2392}S_{77}$). Panel D shows theoretical values of center-mass variance calculated by the method presented in this paper. Center mass variance for most abundant peaks for Bovine serum albumin (the most abundant aggregated variant has 42 additional neutrons in comparison to the monoisotopic variant) calculated from exact masses obtained by `isoDalton` software. Of note, this is very important how many fine peaks will be calculated what can be observed when comparing thresholds $100,000$ and $1,000,000$ set in `isoDalton`

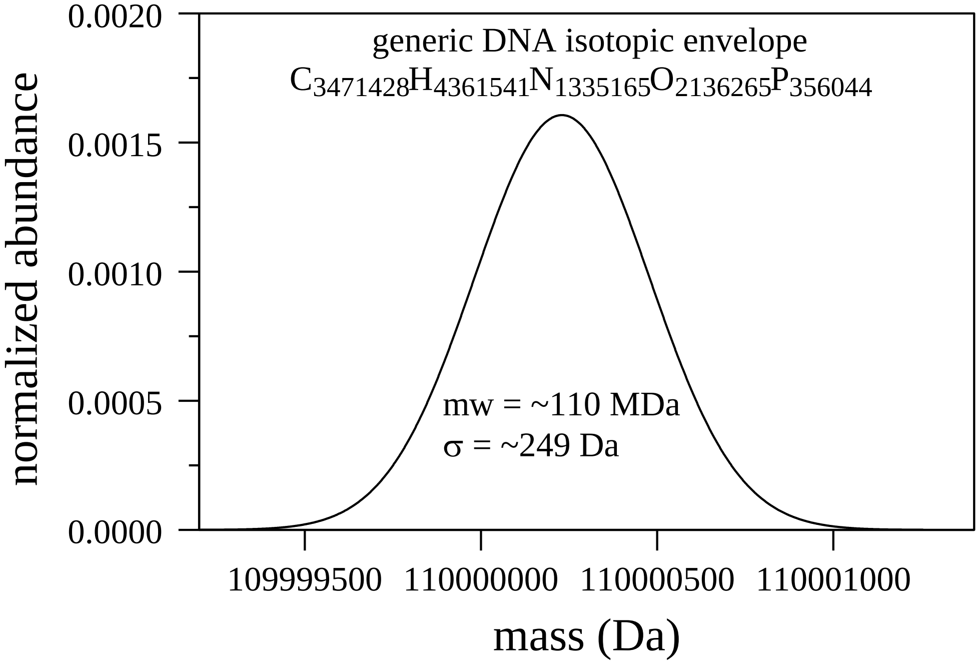

Supplementary Figure 5: This plot illustrates the envelope of the isotopic profile of a generic DNA molecule (composed of equal numbers of A,G,C and T) of molecular weight $\sim$ 110 MDa calculated using FFT-based ultrahigh speed method (see Rockwood and Van Orden, *Ultrahigh-speed calculation of isotope distributions*, Anal. Chem. 68(13):2027–2030, 1996). Normalizing this distribution to unit probability and calculating the standard deviation, the result is 249 Da. The FWHM of the peak is 586 Da.

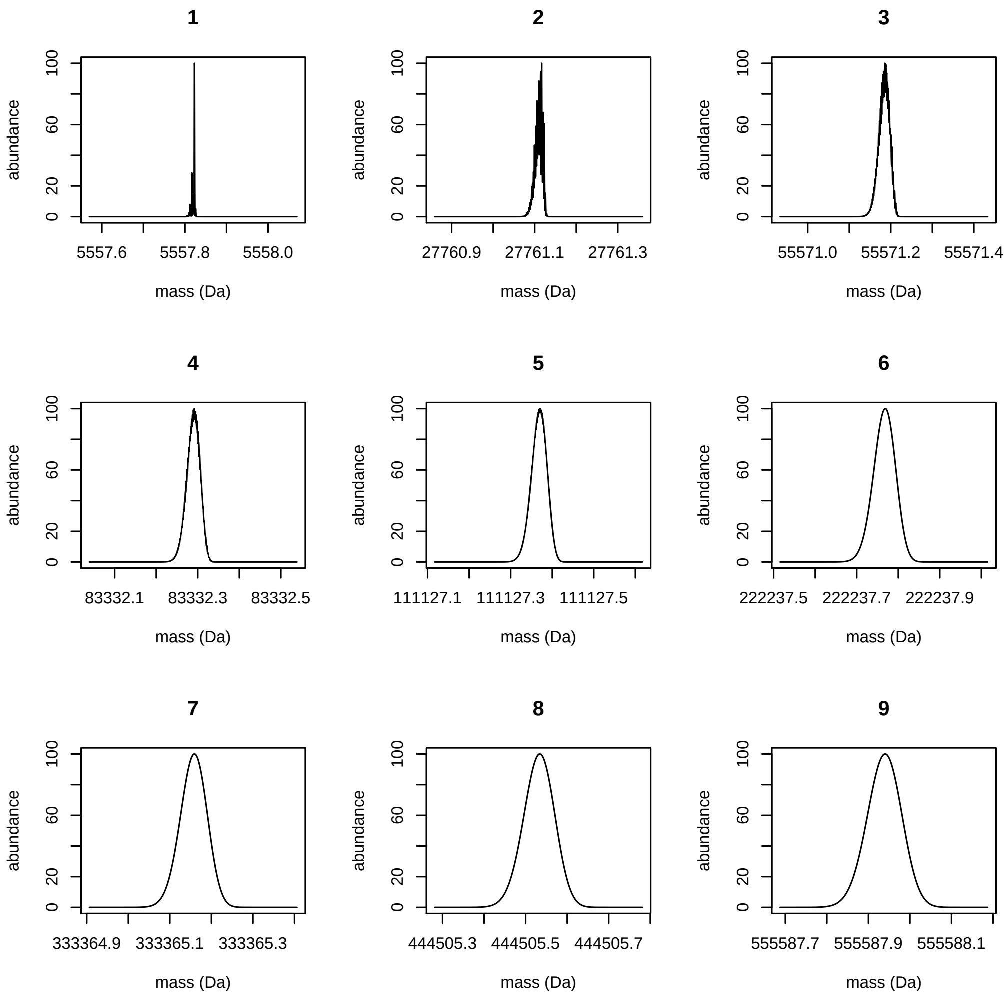

Supplementary Figure 6: The isotopic fine structure for most abundant peak of poly-averagines (for chemical formulas please refer to Supplementary Table 2) obtained by ultrahigh resolution calculations in `mercury` tool for a simulated profile-mode distribution with in which the distribution is convoluted with a narrow Gaussian function and sampled at 2048 points. The isotopic abundances and masses used for this calculation differed slightly from the values used in the rest of this article.

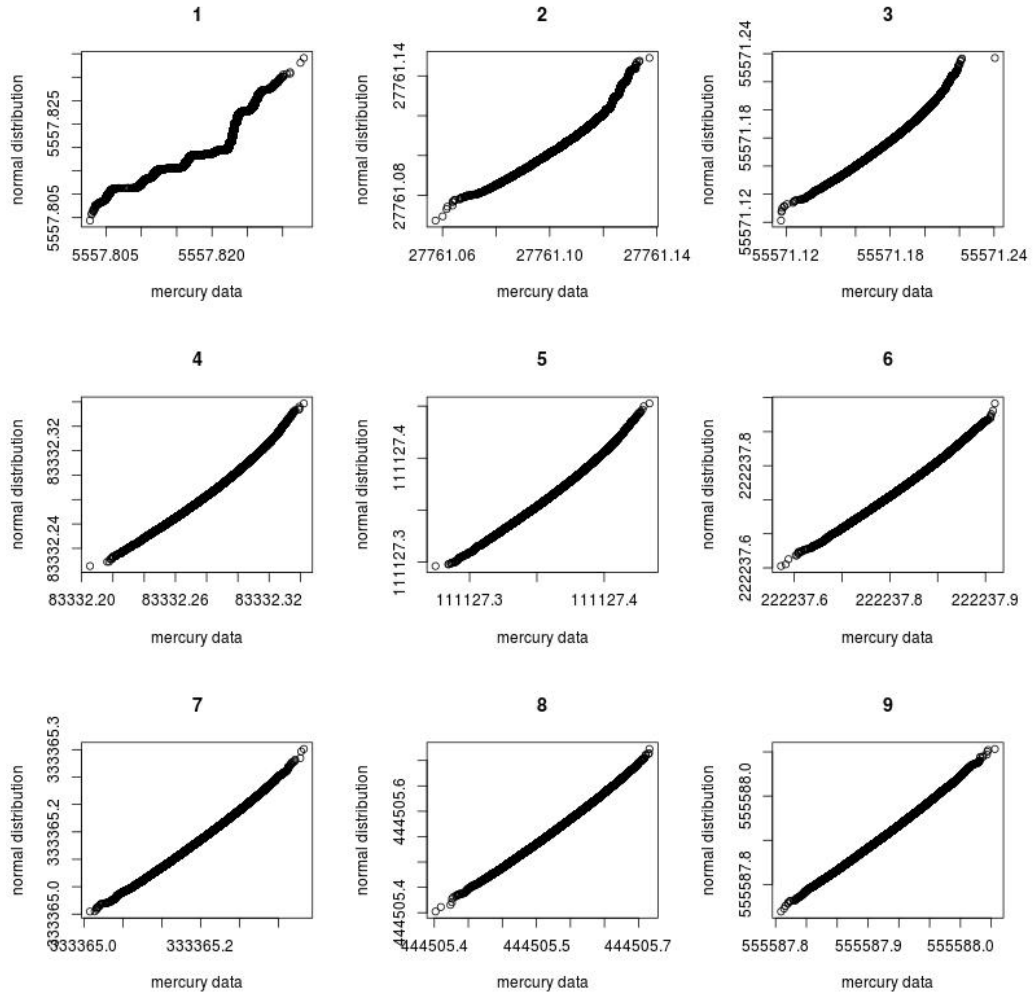

Supplementary Figure 7: QQ-plot showing difference between the fine isotopic distribution of 9 poly-averagines (for chemical formulas please refer to Supplementary Table 2) and corresponding normal distributions. When the molecular weight is large, there is little or no observable difference between the normal distribution and the fine isotopic distribution.
